# Iterative computational and rational design generates diverse PET hydrolases for plastic depolymerization

**DOI:** 10.1038/s41467-026-78471-3

**Published:** 2026-11-03

**Authors:** Brenna Norton-Baker, Benjamin Fram, Nicole N. Thadani, Evan Komp, Yang Su, Olateju O. Storment, Natasha P. Murphy, Samuel Lim, Erika Erickson, Nathan Rollins, Japheth E. Gado, Courtney A. Shearer, Irimpan I. Mathews, Ian Truebridge, Maria Llases, Christopher D. Bahl, Ritimukta Sarangi, Debora S. Marks, John E. McGeehan, Chris Sander, Gregg T. Beckham, Nicholas P. Gauthier

**Affiliations:** 1 Renewable Resources and Enabling Sciences Center, National Laboratory of the Rockies, Golden, CO USA; 2 BOTTLE Consortium, Golden, CO USA; 3 Agile BioFoundry, Emeryville, CA USA; 4 Department of Systems Biology, Harvard Medical School, Boston, MA USA; 5 Department of Data Sciences, Dana-Farber Cancer Institute, Boston, MA USA; 6 Integrated Science and Engineering Division, Underwood International College, Yonsei University, Incheon, South Korea; 7 SLAC National Accelerator Laboratory, Stanford Synchrotron Radiation Lightsource, Menlo Park, CA USA; 8 Institute for Protein Innovation, Boston, MA USA; 9 Dana-Farber/Boston Children’s Cancer and Blood Disorders Center, Harvard Medical School, Boston, MA USA; 10 Present Address: Apriori Bio, Somerville, MA USA; 11 Present Address: Aera Therapeutics, Cambridge, MA USA; 12 Present Address: AI Proteins, Boston, MA USA

**Keywords:** Protein design, Protein engineering, Coevolution

## Abstract

Evolutionary relationships among homologous enzymes can be leveraged to introduce large numbers of amino acid substitutions for improved functional properties. Here, we combine evolution-informed design (EVdesign) with rational design to engineer improved poly(ethylene terephthalate) (PET) hydrolases (PETases). Designs derived from multiple PETases reveal strong parent-dependent differences in success and performance, with *Thermobifida fusca Tf*Cut2 emerging as the most design-amenable scaffold. Increasing sequence diversification reduces the number of active enzymes but broadens performance variance, enabling discovery of high-performing outliers. Four rational mutations overcome a thermostability bottleneck in *Tf*Cut2 and this variant provides a robust scaffold for iterative EVdesign, which further improves activity. Overall, we experimentally test 603 designs from eight parent PETases. More than 400 variants containing 12-68 substitutions were able to depolymerize PET, nearly all outperforming their parent enzymes, and many approach benchmark LCC-ICCG performance in partial conversion assays. These results establish EVdesign as a robust strategy for generating diverse, high-activity enzymes.

## Abstract (web summary)

Here the authors combine computational and rational protein design to generate hundreds of PET-depolymerizing enzymes, many more active than their parent enzymes, and show that iterative design can further improve performance for plastic recycling.

## Introduction

Mismanaged plastic waste is a global problem that, without novel solutions, will continue to have devastating consequences on the environment. Enzymatic depolymerization is increasingly recognized as a promising means to recycle the widely used plastic poly(ethylene terephthalate) (PET) to its constituent monomers^1–3^. However, significant obstacles remain, such as the requirement for expensive pre-processing and pH control when using enzymes for PET recycling in industrial settings^4^. The first PET-depolymerizing enzyme was reported in 2005, with a hydrolase from *Thermobifida fusca* exhibiting enzymatic activity on PET^5^. The discovery of a PET hydrolase in 2016 from *Ideonella sakaiensis*, a bacterium that can assimilate PET building blocks as major carbon sources, further accelerated interest in enzymatic PET degradation, spurring a wide range of discovery and engineering efforts^6,7^. Since then, multiple enzymes capable of PET hydrolysis have been identified from diverse sources and engineered to improve thermostability and catalytic efficiency^2,7–24^. While these enzymes can depolymerize PET under mild reaction conditions, the implementation of industrial enzymatic PET recycling is, among other process-related factors, hindered by the need for pH control and low activity on PET substrates with high crystallinity^4,25,26^.

Adapting PETases to industrial conditions requires optimization of multiple parameters, such as thermal stability, tolerance to pH extremes, and activity across substrate types. This multivariate challenge is often difficult to address experimentally because protein function is highly sensitive to sequence changes and introducing even a single unfavorable mutation can eliminate activity. As a result, most protein engineering strategies for PETases to date have used approaches such as rational and structure-based design^11,17,21,27,28^, which leverage structural and mechanistic knowledge to make targeted substitutions, natural enzyme discovery to find new enzymes likely to catalyze PET deconstruction^8–10^, or directed evolution^12,24,29,30^, which iteratively screens mutagenesis libraries for improved variants, each introducing a relatively small number of mutations at a time. While such campaigns have achieved notable improvements, they also emphasize the effort required to identify beneficial mutations, underscoring the value of approaches that can navigate larger sequence spaces more efficiently.

**Fig. 1:**
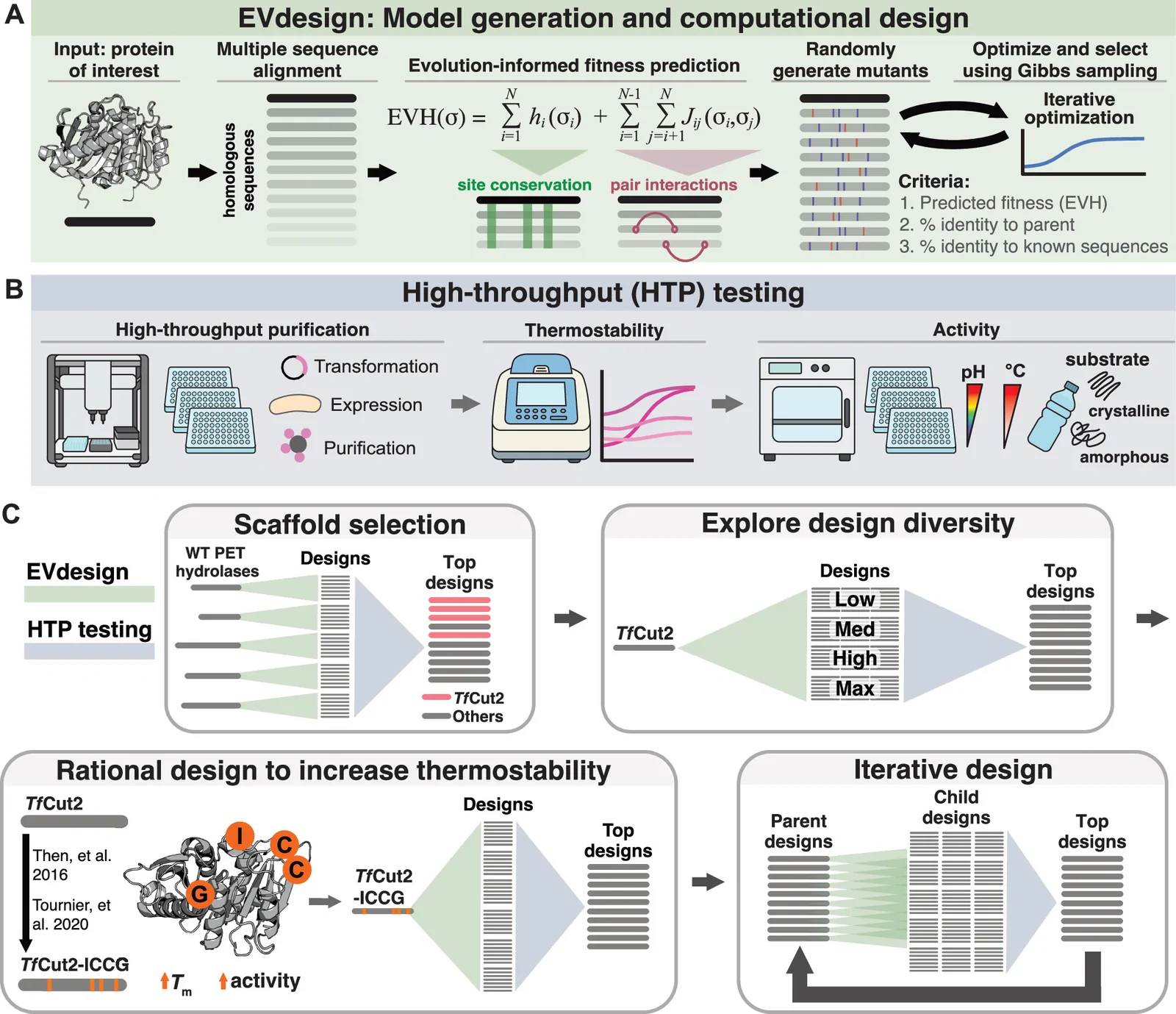
Overview of the design-generation workflow and experimental pipeline. **A** Evolution-informed design (EVdesign) begins with deriving a multiple sequence alignment (MSA) of homologous sequences to a parent protein of interest. Site conservation and pairwise residue interactions are modeled with EVcouplings, and the evolutionary hamiltonian (EVH) is used to estimate the predicted fitness of a specific sequence (*σ*). To derive design sequences, Gibbs sampling is applied by iterative selection of sequences based on an objective function that takes into account the EVH, sequence percent identity to parent, and percent identity to other homologous sequences. **B** High-throughput (HTP) testing of the variants was assisted by an OT-2 robot for enzyme expression and purification^39^. Purified enzymes were evaluated for thermostability by differential scanning fluorimetry (DSF) and for enzymatic activity on amorphous and crystalline PET substrates across a range of pH and temperature conditions. **C** Initial EV design rounds were performed on a panel of natural PET hydrolases to identify a robust and design-amenable parent, leading to the selection of *Tf*Cut2. Subsequent rounds expanded design space around *Tf*Cut2 to explore the relationship between sequence diversity and functional performance, yielding increasingly active and thermostable variants. To further enhance this scaffold, four rational mutations (ICCG) were incorporated, generating the *Tf*Cut2-ICCG variant. EVdesign was then applied again on this improved background (i.e., design-on-design) as well as *Tf*Cut2 designs (also modified to contain ICCG) to produce a generation of variants with even higher thermostability and PET hydrolysis activity. This iterative framework integrates rational and evolution-guided strategies to progressively optimize the enzyme.

Computational protein design methods offer a path to overcome these limitations; however, most existing approaches for PETase engineering remain conservative in the extent of sequence variation they explore^15^^,^^16^^,^^20^^,^^22^^,^^23^^,^^31^. In contrast, evolution-informed design (EVdesign) is well-suited for generating highly mutated yet functional variants^32^. This design strategy, which is enabled by evolutionary sequence models like EVcouplings and direct coupling analysis^33–35^, exploits patterns in the co-variation of amino acids from large multiple sequence alignments (MSAs) of homologous proteins to identify residues that can mutate together without disrupting structure or activity. The statistical energy formulation that underlies these models (evolutionary hamiltonian or EVH, Fig. 1A), which accounts for both sitewise conservation and pairwise interactions between amino acids, has been shown to accurately predict the effects of mutations^33,34,36^ and has been applied successfully as an objective function for sampling algorithms that explore sequence space for protein design^32,36^. Incorporating these co-evolutionary constraints into design enables the introduction of many simultaneous mutations, allowing exploration of sequences that may not be readily accessible through local mutational searches while also potentially combining multiple beneficial traits into a single variant ^32,36^.

Characterizing enzymatic activity on solid polymer substrates such as PET poses additional challenges, particularly when evaluating large libraries of designed PETases. Although several clever screening strategies have been developed, including crude lysate assays^12^, split-GFP-based activity readouts^30^, and secretion-dependent screens^37,38^, these approaches typically sacrifice either quantitative accuracy or biophysical detail for throughput. Concentration-normalized activity measurements and complementary biophysical characterization, which generally require purified protein, are of paramount importance for accurate assessment of PETase properties. We recently described a semi-automated expression and purification platform^39^ that enables biochemical analysis of hundreds to potentially thousands of PETase variants, bridging the gap between rapid, but low-resolution screens, and fully purified, but low-throughput mechanistically informative assays.

In this work, we use EVdesign to generate a large set of functional PETase variants with high mutational loads, aiming to explore broad sequence space in order to both identify high-performing enzymes and establish a diverse cohort of active scaffolds with distinct functional profiles. We combine this approach with rationally selected mutations previously shown to enhance PETase performance^11^, and evaluate the resulting enzymes across conditions relevant to PET depolymerization, including different PET substrates, temperatures, and pH. This integrated strategy reveals how scaffold choice, mutational load, and iterative design influence performance and yields a large and diverse set of PETase variants with improved activity and stability.

## Results

The EVdesign workflow leverages co-variation patterns from homologous proteins to propose coordinated sets of mutations to a chosen parent enzyme (Fig. 1A). Variants were produced and evaluated using a high-throughput (HTP) expression, purification, and screening pipeline^39^ assisted by a liquid-handling robot that enabled the parallel assessment of thermostability and PET hydrolysis activity across multiple conditions critical for industrial-scale processes, including different substrates (amorphous and crystalline PET), pH conditions, and temperatures (Fig. 1B). Initial design rounds were performed across several extant PET hydrolase scaffolds to identify a robust and design-amenable starting enzyme, leading to the selection of *Tf*Cut2 for subsequent rounds of expanded and iterative design (Fig. 1C).

### Assessing suitable parent enzymes for PETase design

**Fig. 2:**
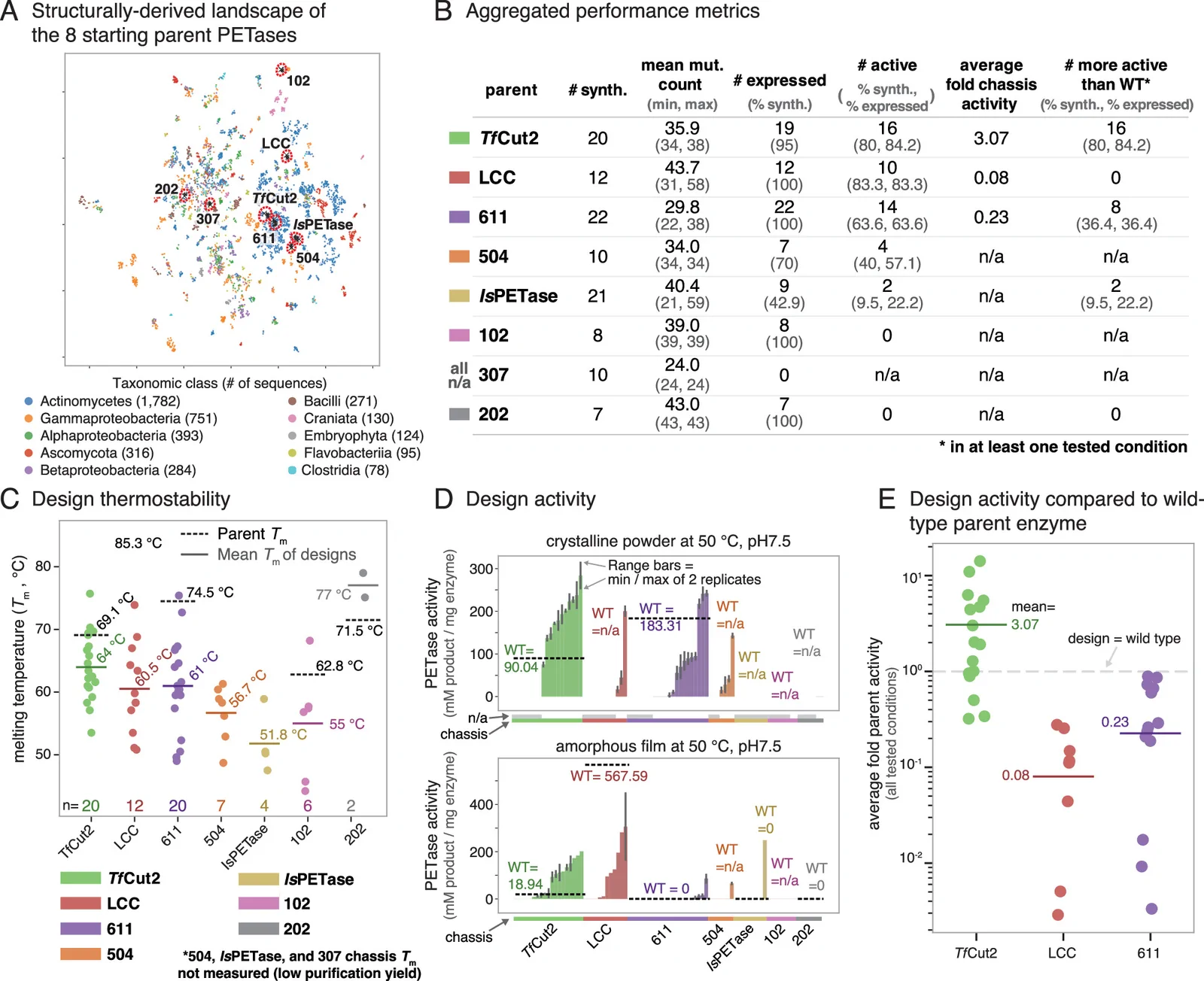
Experimental characterization of designs based on eight wild-type PETase enzymes. **A** Two-dimensional space representation (UMAP^60^) of parent enzymes and designs (structures predicted by AlphaFold 3), and structurally similar proteins in AlphaFoldDB as identified by Foldseek^48,49,61^. Each point is a protein and axis units are arbitrary. Local Distance Difference Test (LDDT) scores between every pair of structures were converted to a distance metric by -log(0.1 + LDDT) for the UMAP visualization. Colors: taxonomic class of the protein’s annotated organism. **B** Summary of design statistics and performance metrics for designs from each scaffold. **C** Thermostability (*T*_m_*T*_*m*_, *y*-axis) determined by DSF. Each dot represents a single design (one replicate). The mean of each design set is indicated by the solid line in the same color, and the *T*_m_*T*_*m*_ of the parent enzyme is shown as a dashed black line. **D** PET-hydrolyzing activity in crystalline powder at 50 °C, pH 7.5 (top) and on amorphous film at 50 °C, pH 7.5 (bottom). Bars represent the mean of two biological replicates or a single replicate for low yield designs. All designs were tested in the prioritized condition (bottom panel); only designs with sufficient expression were tested in the top panel (gray indicator under plot indicates N/A values). Colors indicate parent PETase, which are grouped on the *x*-axis and designs are then sorted by average activity. Range bars: extend from the minimum and maximum values of two replicates. **E** Average fold activity of each design to their parental enzyme was computed on the mean of up to 16 individual conditions (2 replicates per condition for design and parent, or one replicate for low yield designs) and included all conditions in which both parent and design were tested, and for which the parent had measurable activity (avoiding a division by zero, *n* = 19, 12, 21 designs for *Tf*Cut2, LCC, and 611, respectively). Log scale, which is used for clarity of the wide data span, prohibits visualization of designs with zero average fold activity (*n* = 3, 4, and 9 designs for *Tf*Cut2, LCC, and 611, respectively). Color and *x*-axis position indicate parental enzyme. Source data are provided in the Source Data file.

To select a starting enzyme that could tolerate extensive sequence diversification, we initially applied EVdesign to multiple known PETases to evaluate their design compatibility: *Is*PETase^6^, *Tf*Cut2^40^, LCC^41^, and, from our previous work sourcing thermotolerant PETases, 611 (*Sf*Cut), 504, 102, 307, 202^8^. Because the biophysical features that confer design tolerance are not well understood, we explored enzymes that were (a) sequence and/or structurally diverse, (b) exhibited verified PET hydrolysis activity, and (c) were experimentally tractable and would likely be amenable to HTP expression and purification. These starting proteins were diverse when compared with each other (Supplementary Table S1) as well as structurally when compared to their homologs in a two-dimensional landscape (Fig. 2A).

The same EVdesign process was applied to each of the eight starting enzymes to generate designs for gene synthesis and experimental testing: (1) homologous sequences were collected and aligned as a MSA^42^. (2) Next, we generated global probability EVcouplings models of positional and pairwise dependencies from several MSAs (adjusting depth and coverage using the bitscore parameter, “Methods”). The predictive accuracy of each model was assessed by comparing pairwise couplings with known structural contacts, and the model selected for design was chosen to maximize coverage across the entire sequence while also having reasonable predictive accuracy (>50%). (3) Designs were generated by sampling sequences that maximize the model-predicted fitness (evolutionary hamiltonian or EVH, Fig. 1A) subject to sequence identity constraints to the parent enzyme and other designs being generated in the same batch (“Methods”). Depending on the parent enzyme, 5–20% of amino acids in the protein were mutated, which resulted in an average of 24 to 44 mutations per design (Fig. 2B).

The designs were subsequently synthesized, purified and tested using our previously reported HTP production platform^39^. Briefly, genes were codon-optimized for *Escherichia coli* and synthesized commercially, cloned into the pCDB179 vector, which conferred an N-terminal His- and SUMO tag. The resulting plasmids were transformed into chemically competent *E. coli* and expressed in autoinduction media in well plates, with expression and purification assisted by a liquid-handling robot (Opentrons OT-2). After expression, the cells were lysed with a detergent, and Ni-charged magnetic beads were introduced to bind the His-tagged PETase variants. The magnetic beads were washed, and SUMO protease was introduced to liberate PETase variants, yielding a tagless product. Protein yield was quantified with BCA assay and, for samples with measurable yields, the concentration was adjusted to 0.3 mg/mL for high-yield variants or 0.1 mg/mL for low-yield variants. Thermostability was then evaluated using differential scanning fluorimetry (DSF) with Sypro Orange as an unfolding reporter dye.

Activity was assayed against two commercially available PET substrates, an amorphous film (aFilm; 4.0 ± 2.0% crystallinity) and a crystalline powder (cryPow; 39.3 ± 2.0% crystallinity), characterization of which has been previously reported^8^. Reactions were performed across pH 4.5–8.5 (with ranges varying by experimental round) at 50 °C and 70 °C. Concentration-normalized enzymes were grouped by yield and tested in as many conditions as allowable by yield limitations. Depending on total amount (μg) of enzyme available, enzymes were tested in all 16 conditions (2 substrates, 4 pHs, 2 temperatures) in duplicate (32 total conditions), 8 conditions with 1 replicate (1 substrate, 4 pHs, 2 temperatures), or 4 conditions with 1 replicate (1 substrate, 4 pHs, 1 temperature). Enzymes were introduced at a final loading of 1.43 mg/g PET with a substrate PET solid loading of 0.7 wt/wt%. The reactions were incubated for 48 h, and the reaction progression was measured by UV-vis spectroscopy of the terephthalic acid (TPA), mono(2-hydroxyethyl) terephthalate (MHET), and bis(2-hydroxyethyl) terephthalate (BHET) released via enzymatic depolymerization.

Although all of the designs had increases in predicted fitness (EVH, Supplementary Fig. S1), which has been previously reported to correlate with thermostability^32–35^, most of these designs showed decreases in melting temperature, the magnitude of which varied depending on parent enzyme (Fig. 2C). This loss of thermostability was also surprising as it was different from what we previously observed when applying a similar EV design strategy to the TEM-1 *β*-lactamase enzyme^32^. The melting temperatures of the parents that we were able to measure were quite high (Fig. 2C), and it is possible that further increases require a different approach than the design methodology applied here.

The designs as well as the parents themselves had a wide range of activity profiles, and some of the designs were considerably more performant than the parent from which they were derived (Fig. 2D, and Supplementary Fig. S2). For the *Tf*Cut2-derived designs in particular, many of the purifiable designs had large increases in activity (mean 3-fold and max 14-fold increase, Fig. 2E), and every one of the 16 active *Tf*Cut2-derived designs was more performant than the parent in at least one condition. It is important to note that, due to a data entry error, the *Tf*Cut2 parent we characterized in this round contained a single extra amino acid (tyrosine) at the C-terminus of the synthesized and tested protein; however, this version of the enzyme performed similarly to the corrected wild type used in subsequent experiments (Supplementary Fig. S3). Several of the 611-derived designs were frequently more active than wild type, but on average, the designs exhibited about 1/3 of the parent activity. Although several of the LCC-derived designs had high absolute activity in a few of the conditions (especially towards amorphous film at 50 °C, Supplementary Fig. S2), no design outperformed the LCC parent in any condition (maximum 0.54-fold LCC activity in a single condition). Overall, five of the eight parents (504, 611, *Is*PETase, LCC, and *Tf*Cut2) had designs that showed measurable activity (Fig. 2D, and Supplementary Fig. S2), while designs originating from the other three parents were either tested and had no measurable activity (102 and 202), or they were unable to be purified and were therefore not tested (307). Notably, we were unable to obtain sufficient soluble protein for activity testing from three of the parent enzymes (102, 307, and 504). In a previous study, these enzymes were successfully purified and were found to depolymerize PET^8^. In that study, the purification of each enzyme was performed at a much larger scale, indicating that the low yield we observed is likely due to the low-volume, high-throughput purification workflow. In addition, although *Is*PETase and 202 were subjected to activity testing, we did not detect measurable activity under our assay conditions; this outcome is consistent with prior reports showing minimal or no activity for these enzymes at the elevated temperatures used here ^8^.

Throughout this work, we also generated and tested ~140 additional designs that served as internal controls, explored alternative design strategies (such as upweighting specific positions during Gibbs sampling), and explored additional published PETase scaffolds such as HotPETase^12^ and LCC-ICCG^11^. Although these designs and controls are not highlighted in the main manuscript, a short description is provided in Supplementary Note S1, and the corresponding data are included in Supplementary Data 1 as a community resource.

The fraction of active designs with increases in thermostability and activity compared to their parents was encouraging, particularly for *Tf*Cut2-based designs. We thus decided to move forward primarily with *Tf*Cut2 as the parent of choice as designs generated from it (1) had the highest increase in activity relative to parent (average of ~3.1-fold increase, and every active design was better in at least one condition) (Fig. 2D, and Supplementary Fig. S2), (2) had some of the highest absolute activity measurements, (3) had some of the highest average melting temperatures (~64 °C, decreasing ~5.1 °C compared to parent), and (4) were likely to be purifiable and active, potentially yielding a higher percentage of active enzymes.

### The effect of design set diversity on enzymatic thermostability and activity

In our second round of design, we focused on *Tf*Cut2, and explored whether varying the amount of diversity introduced during design generation affects the thermostability and activity properties of the resulting designs.

**Fig. 3:**
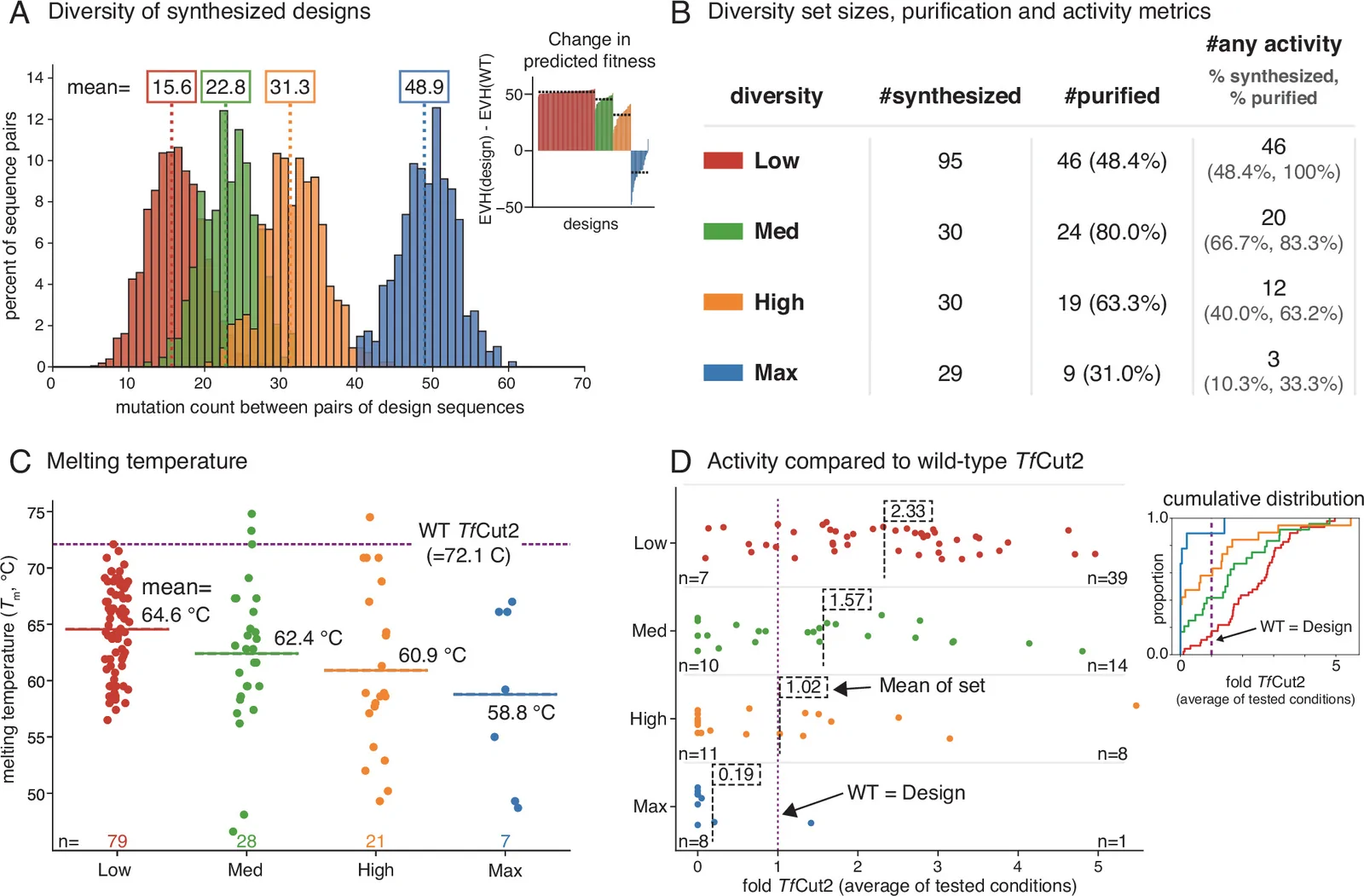
The magnitude of diversity in a design set impacts the fraction of active enzymes, as well as the thermostability and activity profiles. Colors indicate the design set diversity level (red=low, green=medium, orange=high, blue=maximum diversity). **A** Design sets were generated by varying the number of iterations and convergence rate Gibbs-sampling parameters, which resulted in varying intra-design set sequence identity. A histogram of intra-design mutation counts (all designs vs. all designs) is shown for each of the 4 design sets. The mean of each is indicated in a colored box and dashed line. Inset plot depicts the change in predicted fitness relative to wild type (ΔEVH) for each diversity set (dashed line = mean ΔEVH of set). **B** Statistics of the number of designs tested, the number purified (i.e., above concentration quantification threshold), and the number with measurable activity in each design set. **C** The melting temperature (*T*_m_*T*_*m*_) of each design grouped by design set. All purification wells were evaluated by DSF, which was sufficiently sensitive to yield measurable *T*_m_ *T*_*m*_ values for some designs below the purification concentration threshold. Each dot is a single design. The mean *T*_m_ *T*_*m*_ of each design set is indicated with a solid line, and the *T*_m_ *T*_*m*_ of the parent enzyme (*Tf*Cut2) is shown with a purple dashed line (72.1 °C). **D** Average activity (across up to 16 conditions) of each design compared to the original parent (*Tf*Cut2). The average is computed across all conditions (average design divided by average parent) in which both the design and parent were tested, and the parent was not zero (to avoid a division by zero). Average computed from two replicates, or a single replicate if expression was low. The number of designs (*n*) less than and greater than wild type *Tf*Cut2 for each set are indicated to the left and right of the dotted purple line, respectively. Source data are provided in the Source Data file.

Sequence diversity of the designs can be computationally tuned by several parameters, including adjusting the Gibbs sampling parameters and/or adjusting the design objective function (e.g., design sequence identity to wild type or other designs). Four groups of designs with differing amounts of intra-design set diversity were generated on the *Tf*Cut2 scaffold by varying Gibbs sampling parameters related to the convergence rate and number of sampling iterations. The mutation count for all designs was set to 10–15% of the sequence length (26–39 mutations out of 261 amino acids). The final four synthesized design sets, henceforth referred to as Low (low diversity, *n* = 95), Med (medium diversity, *n* = 30), High (high diversity, *n* = 30), and Max (maximum diversity, *n* = 29), contained the expected intra-design diversity as assessed by pairwise mutation counts (Fig. 3A); Low, Med, High, and Max had an average of 15.6, 22.8, 31.3, and 48.9 mutations between the other designs in the set, respectively. Importantly and as expected, this increasing diversity came at the expense of predicted fitness (Fig. 3A, inset). We successfully purified and characterized 98 of the 184 designed variants (Fig. 3B) using the same HTP experimental pipeline described above.

Although a small number of designs exhibited melting temperatures above the already thermostable *Tf*Cut2 (*T*_m_*T*_*m*_ = 72.1 °C), most showed reduced thermostability overall (Fig. 3C). Within the design sets, however, the predicted evolutionary fitness correlated with thermostability: namely, the average *T*_m_ *T*_*m*_ decreased as the diversity increased, with the Low, Med, High, and Max diversity sets ranking from highest to lowest average *T*_m_. At the same time, the variance in *T*_m_ *T*_*m*_ increased with diversity (Low=15.5 °C^2^, Med=41.4 °C^2^, High=54.8 °C^2^, Max=63.4 °C^2^), indicating that highly diversified sets contained a broader range of outcomes. Notably, the three most thermostable variants did not arise from the Low-diversity set, but from the Med and High sets. These results suggest that increasing sequence diversity–even at the expense of average thermostability–can expand the search space and enable the discovery of high-performing outlier variants.

Even with decreased thermostability, many of the characterized designs exhibited higher activity than *Tf*Cut2, and there was a clear relationship between the diversity of the design set and how often and by what magnitude they outperformed parent activity (Fig. 3D, and Supplementary Fig. S4). The less diverse the sequence set was, the higher the average activity, i.e., the Low, Med, High, and Max diversity sets had 2.33-fold, 1.57-fold, 1.02-fold, and 0.19-fold the activity of wild type, respectively (Fig. 3D). Similarly, diversity level was also associated with the percent of conditions in which the designs outperformed wild-type *Tf*Cut2, with the Low, Med, High, and Max diversity sets demonstrating on average higher activity than *Tf*Cut2 in 70%, 49%, 30%, and 8% of the conditions in which they were tested, respectively (Supplementary Fig. S5). However, individual high-performing outliers may be overlooked by focusing on the design set average activity. This distinction is important because, in many protein engineering campaigns, the primary objective is not to improve the mean performance of the design set, but to discover rare variants with exceptional activity.

Interestingly, the most performant design, which had an average activity increase of 5.4-fold relative to *Tf*Cut2,and was the top-ranked design in 10 of 16 conditions, came from the second most diverse (High) design set. In addition, two Med designs were also ranked as top performers (3rd and 6th highest average fold activity of parent, Fig. 3D). Notably, both diversity sets had fewer designs (30 versus 95), and their designs, on average, performed worse than in the Low set. As with the thermostability results, the variance for the average activity versus parent was increased in the Med (1.5-(fold *Tf*Cut2)^2^) and High (2.1-(fold *Tf*Cut2)^2^) sets relative to the Low (1.4-(fold *Tf*Cut2)^2^) diversity set. Together, these results indicate that increasing sequence diversity broadens the performance landscape, enabling the discovery of rare, high-performing outliers even when the average activity declines. Thus, if the goal of a design campaign is to identify exceptional top performers, relaxing sampling constraints to increase diversity may be advantageous. Conversely, if the objective is to maximize the number of reliably improved designs, prioritizing higher predicted fitness (and therefore lower diversity) may be more effective.

Overall, we observed increases in activity and decreases in thermostability in a large fraction of the designs. Encouragingly, in this single-shot design round that introduced ~25–40 mutations per sequence, the average activity levels across conditions for many of the designs was 4- to 5-fold higher than *Tf*Cut2. The number of performant designs and the magnitude of activity compared to wild type varied based on the experimental testing condition and were clearly related to the diversity level. Due to the overall average increases in performance as well as throughput constraints (i.e., activity testing is labor-intensive and synthetic genes are costly), we decided to move forward with sampling parameters that were used to generate the Low dataset.

### Rational engineering improves thermostability and activity and produces a robust parent scaffold for EV design

We were encouraged by the overall percentage of designs that were active and the magnitude of increase over the parental (wild-type) *Tf*Cut2. However, these designs were generally less active than benchmark enzymes like LCC-ICCG^11^. Unlike the high melting temperature of LCC-ICCG (~93 °C), over 91% and 96% of the diversity dataset designs (see previous section) had melting temperatures below the 70 °C condition in which many of the assays were performed and below the 72  °C temperature reported to maximize kinetic turnover of LCC-ICCG^11^, respectively. The four ICCG substitutions (F243I, D238C, S283C, and Y127G) introduced into LCC were previously shown to substantially increase thermostability and improve PET conversion by 30% in bioreactor conditions^11^. Motivated by these observations, we next evaluated whether introducing the equivalent ICCG substitutions into *Tf*Cut2 and EVdesign variants could similarly enhance stability and activity.

**Fig. 4:**
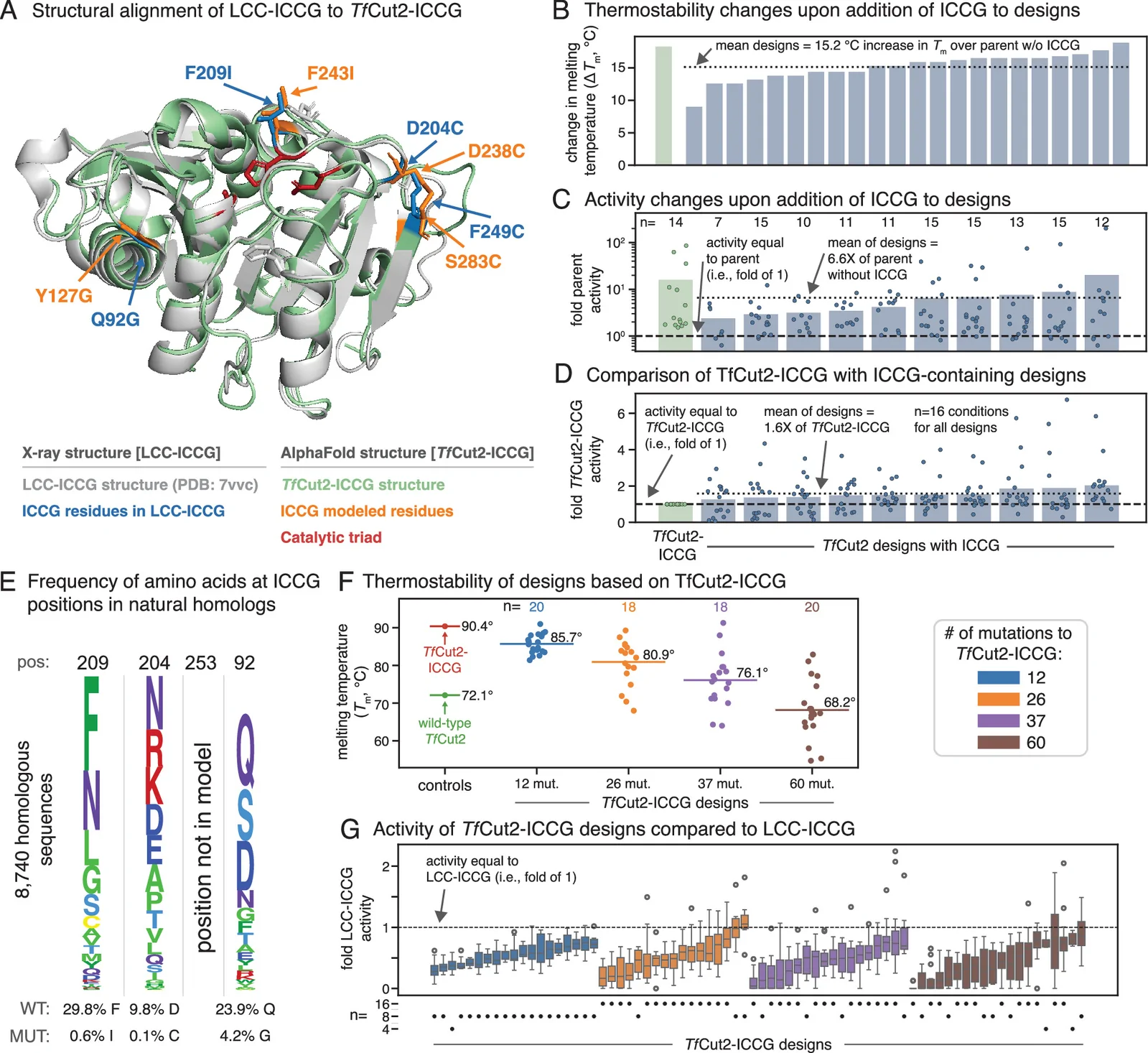
Incorporation of the ICCG substitutions into *Tf*Cut2 increases thermostability and activity, and establishes *Tf*Cut2-ICCG as an improved scaffold for EV design. **A** Alignment of AlphaFold-predicted *Tf*Cut2-ICCG structure to X-ray structure of LCC-ICCG (PDB: 7VVC)^18^. LCC-ICCG is shown in gray with ICCG residues in blue. *Tf*Cut2-ICCG is shown in green with the catalytic triad shown in red and ICCG residues in orange. **B** Change in thermostability upon ICCG addition to *Tf*Cut2 (green) and 22 *Tf*Cut2-based designs (blue). The gray dashed line indicates the mean *T*_m_ *T*_*m*_ increase of 15.2 °C. **C** Fold activity of ICCG-containing variants compared to their parent (Validation Set, “Methods”). Each dot represents the fold of parent in a single condition; bar height indicates the average across conditions in which both variants were tested and the parent had measurable activity. Mean of all designs is indicated with the dotted line. **D** Fold activity of designs and *Tf*Cut2 to *Tf*Cut2-ICCG. Dots and bars as described in (**C**), with the averages calculated relative to *Tf*Cut2-ICCG rather than individual parents. **E** Logo plots of ICCG positions in *Tf*Cut2-based multiple sequence alignment containing 8740 homologs, showing frequencies of wild-type and ICCG amino acids. **F** Thermostability of *Tf*Cut2-ICCG-derived designs. Each dot represents one design; colors indicate mutation count (blue = 12, orange = 26, purple = 37, brown = 60, green = *Tf*Cut2, red = *Tf*Cut2-ICCG). **G** Activity of *Tf*Cut2-ICCG based designs relative to LCC-ICCG. Fold computed as in (**C**) but with LCC-ICCG rather than each parent. Box-and-whisker plots display the median (center line), interquartile range (IQR, bounds of box), points within 1.5 × IQR (whiskers), and outliers that extend beyond whiskers (circles). Number of conditions (*n*) used for fold calculation indicated below plots. Colors as in (**F**). Thermostability values are from one replicate; fold activities are based on two replicates, or one for low-expression designs. Source data are provided in the Source Data file.

Structural alignment of the previously reported X-ray structures of *Tf*Cut2 and LCC-ICCG (PDB: 4CG1, chain A, and 7VVC, chain A)^18,43^ showed that the two enzymes share a highly similar backbone architecture (RMSD = 0.55 Å over 1379 aligned atoms; Supplementary Fig. S6). This alignment also enabled the direct identification of the positions in *Tf*Cut2 equivalent to the four ICCG substitutions in LCC-ICCG. The AlphaFold model of *Tf*Cut2 bearing the ICCG substitutions, which positions side chains using molecular dynamics with Assisted Model Building with Energy Refinement, also predicts that these residues adopt similar positions and side-chain orientations to those observed in the crystal structure of LCC-ICCG (Fig. 4A).

We introduced the ICCG mutations - of which the CC pair had previously been reported in *Tf*Cut2^44^ - into wild-type *Tf*Cut2 and 22 of the top *Tf*Cut2 designs. Each of these rational designs was synthesized, cloned, expressed, and characterized as described above. The addition of the four ICCG mutations resulted in large increases in thermostability in *Tf*Cut2 (18.3 °C) and in all designs (min / mean / max = 9.0 °C/15.2 °C/18.9 °C) (Fig. 4B). To compare activity to their parent sequences and *Tf*Cut2-ICCG, each of which were profiled across multiple experimental rounds, we re-expressed, purified, and assayed 10 of these ICCG-addition rational designs as well as 72 other top designs in a single experimental round (Validation Set, “Methods”). In this experimental round, average activity across 16 conditions increased considerably upon the addition of ICCG to *Tf*Cut2 (16-fold increase) as well as for all designs (min/mean/max of 2.4-fold/6.6-fold/20.3-fold) (Fig. 4C). Although *Tf*Cut2-ICCG had one of the highest increases in activity, the *Tf*Cut2-based designs with ICCG all outperformed this rationally-designed version of the *Tf*Cut2 parent (Fig. 4D).

We next explored whether *Tf*Cut2-ICCG was a suitable scaffold for EV design. As the ICCG residues are nearly non-existent in the homolog MSA of *Tf*Cut2 (Fig. 4E), it is possible that EVdesign would not be tolerant of these amino acid substitutions. In this round of design, we also evaluated the impact of mutational burden on performance, generating and analyzing 80 *Tf*Cut2-ICCG designs distributed across 4 mutational load categories (12, 26, 37, and 60 mutations, including ICCG).

Thermostability (*T*_m_ *T*_*m*_) was assessed in all of the *Tf*Cut2-ICCG designs that had sufficient purified protein (76 of the 80). Compared to the parental scaffold *Tf*Cut2-ICCG, the average thermostability of the designs decreased, and the trend of reduced thermostability was again observed with increasing mutational load (Fig. 4F). Importantly, all 76 designs had a *T*_m_ *T*_*m*_ greater than the minimum temperature (50 °C) at which the activity assays were performed, and 59 had T_m_ *T*_*m*_ values that were at or above the maximum assay temperature of 70 °C.

For 74 out of the 80 *Tf*Cut2-ICCG designs, we were able to produce sufficient purified protein to perform activity characterization. All these tested designs retained catalytic function, demonstrating activity in at least one condition (Fig. 4G, and Supplementary Fig. S7). Notably, several *Tf*Cut2-ICCG-derived designs exhibited activity levels comparable to or exceeding those of the benchmark enzyme LCC-ICCG under certain conditions (Fig. 4G, and Supplementary Fig. S7). We also observed a modest relationship between the number of mutations and overall activity–designs with fewer mutations are more likely to be active in all conditions (Supplementary Fig. S8). Interestingly, the relationship between activity and mutational burden was temperature-dependent: at 70 °C, they were inversely correlated (i.e., the higher the number of mutations, the lower the average activity), whereas at 50 °C, this trend was reversed (Supplementary Fig. S9). The inverse correlation at high temperature may stem from mutation-induced destabilization (i.e., reduced thermal stability, Fig. 4F), leading to diminished catalytic performance under heat stress.

Overall, these results suggest that designs built on the *Tf*Cut2-ICCG scaffold retain high catalytic function even at elevated mutational loads, despite the scaffold incorporating rational mutations not found in nature, thus demonstrating the viability of using engineered enzyme parents as starting points for evolutionary design.

### Iterative EVdesign leads to variants with further increases in activity

**Fig. 5:**
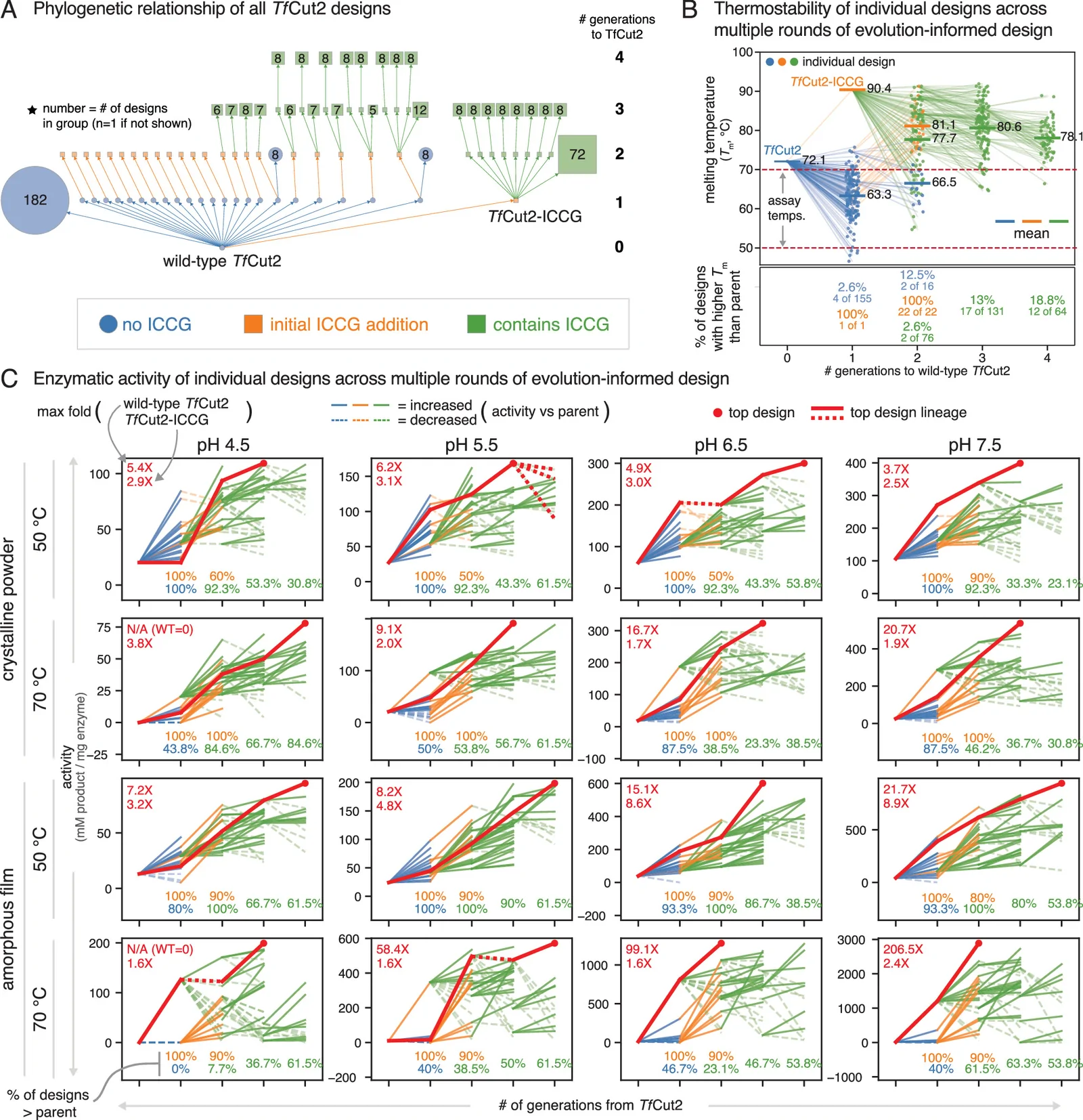
Iterative design enables further sequence diversification and can improve activity, expression, and thermostability. **A** Design lineage of all *Tf*Cut2-derived variants, spanning up to four successive rounds of EVdesign starting from the wild-type *Tf*Cut2. Arrows represent a parent child relationship: orange = addition of ICCG, blue = EVdesign on non-ICCG parent, green = EVdesign on ICCG-containing parent. Blue circles are designs without ICCG. Green squares are designs that contain ICCG. Orange squares indicate the addition of ICCG. Number = design count (*n* = 1 if not indicated). **B** The melting temperature across successive design generations. Each point represents the melting temperature (*T*_*m*_) of a single design (one replicate). Thin connecting lines link each design to its parent, indicating the change in thermostability introduced in that design step. Thick horizontal lines mark the mean *T*_m_  *T*_*m*_ of each generation. Colors correspond to lineage categories defined in (**A**). The percentages below indicate the fraction of designs in each generation that have higher  *T*_m_ *T*_*m*_ than their parent. **C** Enzymatic activity of selected designs measured in a single purification and assay round (Validation Set, “Methods”). Each panel represents one reaction condition. Columns vary in buffer pH; rows vary in substrate type (crystalline powder or amorphous film) and temperature (50 °C or 70 °C). Lines connect design to its parent (solid = increased activity; dashed = decreased, mean activity of two replicates or one if design had low expression). The highest-performing design in each condition is marked in red; red lines trace its lineage. Fold improvements relative to wild-type *Tf*Cut2 (top values) and *Tf*Cut2-ICCG (bottom values) are shown in the upper-left of each panel. Percentages indicate the fraction of designs more active than their parent. Values represent the mean of two biological replicates. Source data are provided in the Source Data file.

We next evaluated whether EVdesign could be iterated, generating designs on top of previously engineered sequences to further diversify in the sequence space while maintaining or improving activity. We selected 19 parent designs for additional design rounds: 11 *Tf*Cut2-based designs (nine of which later received the ICCG substitutions) and eight *Tf*Cut2-ICCG-based designs. For each parent, we applied the EVdesign workflow to produce 7-10 second-generation variants, constraining Gibbs sampling to exclude positions mutated in earlier rounds. The target mutation load was 1–4% of sequence length, resulting in 7-9 additional substitutions per design. After synthesis and experimental characterization, the top-performing designs from this round were subjected to further iterative EVdesign. This process generated a multi-generational design lineage reaching up to four generations from the wild-type *Tf*Cut2 scaffold, including the introduction of ICCG (Fig. 5A), ultimately yielding 201 designs (137 in generation 3 and 64 in generation 4).

Similar to the designs generated directly from *Tf*Cut2 and *Tf*Cut2-ICCG, the iterative EVdesigns showed an overall trend toward reduced thermostability, although a subset of variants exhibited increased melting temperatures (Fig. 5B). On average, the melting temperature decreased by 8.8 °C for *Tf*Cut2 descendants, 12.7 °C for *Tf*Cut2-ICCG descendants, and 4.3 °C for iterative designs. Notably, ~15% of the iterative designs had equal or higher thermostability than their parent, compared with <3% for both *Tf*Cut2 and *Tf*Cut2-ICCG direct descendants. The relatively smaller decreases observed in the iterative designs may in part be due to the lower mutational load from their parent designs compared to previous design sets. Importantly, despite these decreases, the absolute melting temperatures remained high, with iterative designs averaging 78.8 °C, compared to 63.3 °C for *Tf*Cut2 descendants and 77.7 °C for *Tf*Cut2-ICCG descendants.

Analysis of the activity profiles of the top *Tf*Cut2-based designs from a single purification and assay round (Validation Set, “Methods”) showed frequent increases in activity relative to the parent (Fig. 5C). To report a single performance metric for multiple designs, we calculated the percentage of all design-parent pairs across all experimental conditions in which the design had higher activity than the parent (the mean of design activity was greater than the mean of the parent). On average, in 61% of the assays EVdesigns had higher activity than their parent, whereas adding ICCG alone resulted in increased activity in 90.5% of the assays. There was a decrease in performance for EVdesigns based on an ICCG-containing parent: those with and without ICCG had increased activity in 57.7% and 73.0% of assays, respectively. These percentages were relatively consistent across experimental conditions, with a few notable differences. (1) Adding ICCG alone resulted in higher performance on amorphous film compared to crystalline powder (98.2% vs 81.5%), as well as higher activity at 70 °C compared to 50 °C (100.0% vs 81.0%). (2) The EVdesigns tended to have higher performance than their parents at 50 °C compared to 70 °C (73.1% vs 50.5%). Analysis of iterative designs (EVdesigns in generations 3 and 4) revealed lower performance gains than designs based directly on *Tf*Cut2 and *Tf*Cut2-ICCG (EVdesigns in generations 1 and 2), with increased activity compared to their parent in 53.9% and 75.3% of assays, respectively. This reduction in performance may reflect two key differences in the search space explored during iterative design: (1) mutations were restricted to positions not altered in previous rounds, and (2) iterative designs contained fewer mutations overall (mean = 8.1, min = 8, max = 9) compared to first-generation designs (mean = 33.2, min = 22, max = 56). Despite the lower average percentage of designs with activity increases, the highest-performing variants emerged from iterative design: the top design in 14 of the 16 assay conditions originated from generation 3 or 4, highlighting the value of iterative diversification for accessing rare, high-performance outliers.

Although the identity of the most performant design varied in each condition, the top design always outperformed both *Tf*Cut2 and *Tf*Cut2-ICCG (Fig. 5C). Across all conditions, these top EVdesigns had, on average, a 34.5-fold (min = 3.7-fold, max=206.5-fold) and 3.3-fold (min = 1.6-fold, max = 8.9-fold) increase in activity from *Tf*Cut2 and *Tf*Cut2-ICCG, respectively. Because wild-type *Tf*Cut2 showed no detectable activity in the 70 °C, pH 4.5 conditions, fold improvements relative to *Tf*Cut2 in those conditions were excluded from the calculation. These gains over wild-type *Tf*Cut2 and the rationally-designed *Tf*Cut2-ICCG demonstrate the potential of iterative EVdesign to diversify sequence and enhance enzymatic activity.

In 15 of the 16 tested conditions, at least one of the designs matched or exceeded the 48-h product formation of the literature benchmark enzyme LCC-ICCG under identical enzyme loading, buffer, substrate, and temperature conditions (mean activity of the top design in each condition = 1.48X LCC-ICCG, Supplementary Fig. S10). Performance gains were most striking under low pH (4.5) and high temperature (70 °C) conditions: the top EVdesign variant showed ~2.2X higher activity on crystalline powder and ~3.3X higher activity on amorphous film, and more than 24 and 36 distinct designs exceeded LCC-ICCG performance on film and powder, respectively. Although absolute activities at the lowest pH values remained low, improvements under acidic conditions are especially meaningful, as enhanced low-pH performance is a key goal for industrial PET deconstruction processes ^4,25^.

Interestingly, in 11 of the 16 conditions, the top designs were all descendants of the most performant design, *Tf*Cut2_EV1, from the High diversity design set. Two designs in particular dominated these top activity profiles: *Tf*Cut2_EV1_G2, which emerged after a single round of iterative design following ICCG addition, and *Tf*Cut2_EV1_G3, which emerged after two rounds of iterative design following ICCG addition. *Tf*Cut2_EV1_G2 exhibited the highest activity in four crystalline powder conditions (three at 70 °C and one at 50 °C), while *Tf*Cut2_EV1_G3 was the top performer in four amorphous film conditions (three at 50 °C and one at 70 °C). The two designs differed by 11 amino acids and contained 46 and 54 substitutions relative to wild-type *Tf*Cut2, respectively. These 11 mutations, which do not locate in the active site region, are dispersed across the structure and may be involved in long-range interactions important for binding or stability.

Two variants (*Tf*Cut2_EV2 and *Tf*Cut2_EV3), which were among the most active on amorphous film at pH 7.5, 70 °C condition, were successfully crystallized. Despite extensive sequence divergence from *Tf*Cut2 (~17% of the sequence = 46 mutations each), both of these variants had nearly identical 3D structures as the original enzyme (RMSD over all atoms = 0.30 Å and 0.35 Å, respectively). A comparison of these two X-ray structures with *Tf*Cut2 revealed no notable divergences from the canonical fold (Supplementary Fig. S11). This similarity indicates that enzymes generated by EVdesign can maintain the backbone structure of target enzymes despite substantial divergence in sequence.

In summary, iterative EVdesign resulted in increases in activity in almost every condition, producing a large cohort of diverse and performant designs. These results further support EVdesign as a useful method for generating diverse designs with large numbers of mutations while maintaining structure and enhancing function, motivating broader consideration as a general design strategy.

## Discussion

This study demonstrates the effectiveness of EVdesign at exploring the functional sequence space of PET hydrolases. The method proved robust to extensive sequence diversification, yielding active and enhanced variants from multiple PETase parents. *Tf*Cut2 emerged as the most design-amenable sequence, producing a high proportion of active variants and the largest average increases in catalytic performance, and we therefore focused on *Tf*Cut2 in subsequent design rounds. Despite the addition of a large number of mutations (mean = 41.0), most of the *Tf*Cut2-based designs (399 of 513) produced during this work remained soluble and active, and many exhibited improved activity under acidic conditions, which is an important feature for prospective industrial plastic recycling processes. Collectively, these results demonstrate that EVdesign can be applied successfully across diverse starting sequences, and that large mutational leaps can not only be tolerated but frequently result in improved activity.

Performance outcomes differed substantially among the natural parent enzymes, even between scaffolds with high sequence and structural similarity. These differences underscore that designability - the tolerance of a protein sequence and fold to extensive sequence modification - remains poorly understood. Consequently, evaluating a small panel of candidate scaffolds early in a design campaign can identify those that support an increased percentage of functional enzymes. It may also be possible to enhance the designability of scaffolds that are normally not tolerant to high mutational loads by stabilizing them first with methods like consensus design or Protein Repair One-Stop Shop (PROSS)^45,46^. The optimal workflow will depend on the specific engineering objective and available throughput, as both the magnitude of mutational diversity and the choice of parent shape experimental success. For example, the repeated emergence of the *Tf*Cut2_EV1 lineage among the highest-performing variants across conditions suggests that certain sequence backgrounds are particularly well-suited to accommodate and benefit from extensive mutational change. Two descendants of this lineage (*Tf*Cut2_EV1_G2 and *Tf*Cut2_EV1_G3) ranked as the top performers across multiple crystalline and amorphous substrate conditions. Their exemplary performance indicates that improved performance can propagate across successive design rounds and that distant regions of sequence space may be locally expandable to reveal high-performance enzymes.

Deliberately modulating design set sequence diversity may enable design campaigns to be tailored towards particular experimental constraints. We find that tuning the diversity systematically (by adjusting the number of Gibbs iterations and convergence rate) alters outcome distributions - less diverse design sets yield a higher fraction of improved variants with a higher average thermostability and activity, whereas greater sequence diversification expands variance and occasionally produces exceptional outliers. When screening throughput is limited, prioritizing sequences with high predicted fitness (i.e., lower within-set diversity) maximizes the discovery rate of reliable performers. Conversely, broader sampling of lower-predicted-fitness sequences (i.e., higher within-set diversity) can reveal rare, high-performing sequences that may not arise through local searches.

Although several designs displayed increased melting temperatures, the EVdesigns on average had a decrease in thermostability. Predicted fitness has been previously shown to correlate with natural protein stability^32–35^, and we explicitly maximized predicted fitness during sequence generation. This observation may in part be due to the parent being already quite thermostable (the *T*_m_ *T*_*m*_ values of the *Tf*Cut2 and *Tf*Cut2-ICCG are 72.1 °C and 90.4 °C, respectively), but more study is required to understand the effects of EVdesign on already highly thermostable scaffolds. Incorporating complementary predictors, such as empirical Δ*ΔG* estimators or learned thermostability scores, may help bias future design rounds toward sequences that are both active and more thermotolerant.

Introducing the four rational ICCG substitutions into *Tf*Cut2 and its derivatives markedly increased both thermostability and catalytic activity, overcoming the thermostability bottleneck observed during parent testing and diversity evaluation. The resulting *Tf*Cut2-ICCG scaffold retained compatibility with EVdesign, despite these rational mutations being nearly non-existent in homologous sequences of *Tf*Cut2. This result demonstrates the robustness of the approach to incomplete evolutionary information and that rational and statistical design methods can be synergistic rather than mutually exclusive. Conceptually, rational stabilization appears to have opened up performance-rich areas of sequence space, after which evolution-informed sampling can efficiently explore functional diversity within that stabilized background. This combined strategy offers a generalizable workflow for engineering enzymes whose natural sequence diversity is insufficient to capture the desired property landscape.

The successful application of iterative rounds of EVdesign illustrates the method’s capacity for continued refinement. Designs frequently retained or exceeded parental activity, even with more than 20% of the sequence being composed of amino acid substitutions (>50 mutations). The repeated emergence of high-performing designs demonstrates that cumulative rounds of design can further expand sequence space while maintaining functional integrity. In contrast to traditional directed evolution or structure-informed rational design, which progress through small mutational steps, this approach enables larger structured exploration of mutational space guided by evolutionary constraints.

The small-scale 48-h endpoint assays used here enabled the standardized evaluation of depolymerization performance across hundreds of variants and multiple conditions; however, additional studies will be required to resolve the underlying reaction mechanisms in greater detail. Building on these results, future work will incorporate time-resolved kinetic analyses to disentangle the contributions of intrinsic catalytic turnover, enzyme stability, and surface residence dynamics. In addition, characterization of the evolution of polymer properties during degradation, such as changes in molecular weight distribution, crystallinity, chain scission patterns, and the formation of insoluble oligomers, will enable a better understanding of enzyme-substrate interactions at the material level. These efforts will be complemented by evaluation of enzyme performance under process-relevant conditions, such as pH-controlled reactions at high solids loadings and high extents of conversion, to assess performance in industrially relevant settings. A rigorous mechanistic investigation integrating these measurements would require substantially expanded experimentation and represents a distinct future research direction focused on mechanistic understanding of PET depolymerization rather than enzyme discovery and design.

Together, these findings further demonstrate EVdesign as a powerful strategy for engineering enzymes with extensive sequence modification. In this study, we generated hundreds of diverse *Tf*Cut2-based designs, many of which retain activity and a considerable number are competitive with the benchmark enzyme LCC-ICCG in the assay conditions tested here, with the recognition that pH-controlled reactions at high solids loadings and high extents of conversion are ultimately necessary^47^. This large cohort of active enzymes in itself may be useful for plastic degradation, as each individual design has its own property profiles (e.g., distinct robustness or tolerance to substrate or reaction pH, etc.), and application of combinations of enzymes may be an effective means of deconstructing in the complex environments characteristic of real polymer recycling streams. We also show that rational approaches integrate well with this methodology, allowing prior knowledge to enhance particular applications (e.g., increasing activity and substrate specificity) even if these features are not well represented in evolution. Future iterations could integrate additional objectives, such as low-pH activity or expression yield, directly into the design generation process. By enabling the generation of large sets of functional and diverse proteins, this framework can augment or enhance the data required for training next-generation predictors and machine-learning models for enzyme design. The bridging of evolutionary modeling with rational design and experimental validation allows EVdesign to accelerate the creation of robust, high-performance biocatalysts for polymer deconstruction and beyond.

## Methods

### Parent protein landscape determination

To produce the structure space plot in Fig. 2A, we leveraged a structural homolog search followed by alignment. The AlphaFold database in pre-compiled searchable format was downloaded directly from the maintainers of foldseek using the “foldseek databases” command^48,49^. Parent predicted structures were combined into a database of their own using “createdb” and searched (“search” command) against AFDB with arguments “--threads 104 --alignment-type 0 --split-memory-limit 230G -s 7.5 -a --max-accept 5000 -c 0.8 --cov-mode 2 -e 1e-3 --e-profile 1e-1 --cov 0.8 --num-iterations 3 --max-seq-id 0.95”. Resulting hits were filtered by *E* < 1e-10, and query and target coverage both >85%. The predicted structures of the remaining 6746 hits were combined with parent and design structures (predicted by AlphaFold 3) to create a database, which was then searched against itself with the following arguments: “--threads 104 --alignment-type 0 --split-memory-limit 230G -a -e 10000 --exhaustive-search” to get full pairwise alignments. Pairwise Local Distance Difference Tests (LDDT) were retrieved by “foldseek convertalis … –format-mode 0 –format-output query,target,evalue,qlen,tlen,qcov,tcov,nident,lddt,lddtfull” and normalized to the average coverage of both strands since LDDTs are only computed for those residues, e.g., LDDTn = LDDT * (tcov + qcov)/2. Normalized LDDTs were converted to a distance metric via - log(0.1 + LDDTn) to produce a pairwise distance matrix used with UMAP with 15 neighbors and a minimum distance of 1.0; all other parameters were left as default. A software dump of the pipeline, all intermediate results, and the final notebook used to create embeddings is available in Supplementary Code and will be released on GitHub after review.

### Design generation

Design variants were generated in a standardized 2 step process (modifications to the process for specific design sets are described in #3 below):

1. *Model generation*. In this step, we derived an unsupervised model of sitewise and pairwise constraints from homologous sequences of an individual parent. This model was later used to score the “predicted fitness” of potential design sequences. We utilized the open source EVcouplings framework library (available at https://github.com/debbiemarkslab/EVcouplings) to generate models of natural PETases or design parents. For any individual model (distinct parent sequence), EVcouplings gathers homologous sequences of the target parent into a MSA. Each model was learned via regularized maximum pseudolikelihood^33,50^. For any given parent, the depth of the MSA was selected by adjusting the bitscore parameter repeatedly with the goal of maximizing both the coverage (how many residues are included in the model) and the accuracy of structural prediction (overlap of top known structural contacts with predicted residue-residue interactions). Generally, the top model used for design generation had a Neff/L (number of effective sequences divided by the length of the target sequence) of 5-10, but this number varied depending on the particular parent. The output of EVcouplings runs, including the parameters used as well as the MSAs, models, and output metrics are available on Zenodo ^51^.
2. *Derive designs by sampling*. We next applied batched Gibbs sampling to generate individual design instances for synthesis, cloning and testing. Conceptually, the process is as follows:
  - Begin with a completely random sequence (***S***) with the same length as the parent sequence.
  - A single position (***P***) is chosen at random for evaluation and possible mutation.
  - An objective function is evaluated for every possible amino acid at ***P*** (n=20), resulting in 20 values (the conditional probability distribution, ***D***) that indicate how well each sequence is able to maximize the predicted fitness of the entire sequence (the EVcouplings Potts Model, a.k.a. Evolutionary Hamiltonian, EVH), enforce a mutational range to the parent sequence (e.g., 10–15% of positions should be mutated), and enforce a minimum mutational distance to other designs generated in the same batch (e.g., at least 10% of the positions have to contain different amino acids).
  - A single amino acid is selected to persist by sampling proportional to ***D***.
  - The process (b) to (d) is repeated *L* times (*L* = length of the parent) for each batch sweep. The inverse temperature (convergence rate) is annealed on a linear schedule from 0.5 to 10 over 20*L batch sweeps (# of sampling iterations).
  - Standard parameters:
    Overall objective function weights (sum should equal 1):
      –energy_weight: Weight given to predicted fitness in the cost function (i.e., EVH, default is 0.34)
      –seq_div_weight: Weight given to sequence diversity in the cost function (default is 0.66). Subweighted as follows (sum of the 3 subweights should equal 1):
        ⁎seq_div_ref: Relative weight of distance to reference (generally set to 0.5)
        ⁎seq_div_gs: Relative weight of distance to other design sequences (generally set to 0.5)
        ⁎seq_div_msa: Relative weight of distance to other sequences in the MSA (generally set to 0)
    •Distance to reference sequence and other design sequences:
      –hamming_ref_seq_min: Minimum distance to reference sequence. This value varied, but was generally between 0.01 and 0.2 (minimum of 1% or 20% of positions mutated).
      –hamming_ref_seq_max: Maximum distance to reference sequence. This value varied, but was generally between 0.04 and 0.25 (maximum of 4% or 25% of positions mutated).
      –hamming_inter_min: Minimum distance to other designs being generated in the same batch, as well as to other sequences in the MSA. This value varied between runs, but generally set to about half of the hamming_ref_seq_max parameter (e.g., 0.05 = designs must be at least 5% different between one another and all sequences in the MSA).
    Miscellaneous:
      –sites_to_ignore: List of positions that are fixed / unavailable to mutate. This parameter was used to fix the ICCG mutations and parent mutations during iterative design.
      –ref_seq_dist_penalty: The cost of reference sequence violations, i.e., hamming distance to sequence is less than hamming_ref_seq_min or more than hamming_ref_seq_max. Generally set to -1000.
      –hamming_cost: The cost of hamming_inter_min violations. Generally set to -10.
      –init_beta_temp: the initial annealing temperature.
  - Code to execute Gibbs sampling is available in Supplementary Code and is available on GitHub ("Code availability").
3. *Modifications to specific design sets:*
  - Design generation is considerably slower (difficult to parallelize) when applying intra-design similarity constraints, which require calculating intra-design similarity between all designs at every step of Gibbs sampling. In order to speed up and parallelize design generation, in the scaffold selection section and the diversity section we generated 1000 designs in 20 batches of 50 designs (each batch was intra-design distance constrained), and then applied hierarchical clustering (scipy function = linkage, distance method=hamming linkage method = ward) to the resulting designs and scipy’s fcluster as a means to choose a diverse, unique set of designs for experimental followup.
  - For the diversity study (Section 2), the annealing schedule and the number of sampling iterations were adjusted to vary the diversity of the Med sample set (number of sampling iterations = 10*L), High sample set (annealing schedule of 0.33 to 10 and number of sampling iterations = 10*L), and Max sample set (annealing schedule of 0.2 to 10 and number of sampling iterations = 10*L) datasets. This was done by setting init_beta_temp to 0.2 and 0.33 (default for other designs was 0.5).

### High-throughput experimental testing

#### Protein expression and purification

Genes were synthesized and cloned into pCDB179 (gifted to Addgene by Christopher Bahl, #91960) by Twist Biosciences. The variants were expressed and purified using a high-throughput, robot-assisted platform^39^. The plasmids were transformed into C41(DE3) *E. coli* cells and expressed in 2 mL autoinduction cultures in 24-deep well plates at 37 °C for 2 h then 18 °C for 40 h. Cells were harvested by centrifugation and lysed via shaking for 1 h in 1.5 mL of 20 mM TRIS pH 8.0, 300 mM NaCl, 5 mM imidazole, 1% n-octyl *β*-D-glucopyranoside supplemented with 0.1 mg/mL DNaseI and 1 mg/mL lysozyme. Each well received 70 μL of a 25% slurry of Ni-charged magnetic beads (Genscript L00295) and binding proceeded for 2 h shaking. The supernatant was then discarded and the magnetic beads washed. To cleave the target protein from the magnetic beads, 22 μg of His-tagged Cth SUMO protease (produced in-house) was added to each well and the cleavage was allowed to proceed for 4 h at room temperature while shaking. The supernatant was then transferred to a fresh plate and a 10 μL aliquot used for quantification via a Pierce Rapid Gold BCA Protein Assay Kit. Concentrations were adjusted via dilution with 20 mM TRIS pH 8.0, 300 mM NaCl to 0.1 mg/mL or 0.3  mg/mL if adjustment to 0.1 mg/mL would exceed the well maximum volume. If concentrations were below 0.1 mg/mL, no dilution occurred.

#### Differential scanning fluorimetry (DSF)

DSF was performed on a Bio-Rad CFX96 Touch Real-Time PCR instrument with the following program: 1. 25 °C: 0:15; 2. 25 °C: 0:31; 3. 25 °C: 0:15 (+0.3 °C/cycle, ramp 0.3 °C/sec); 4. Plateread, 5. Go to 3, 250X. The samples contained 0–0.3 mg/mL protein with a 1X final concentration of Sypro Orange Dye. The melting temperature (*T*_*m*_) was determined as the temperature at the maximum of the first derivative. All curves were manually checked for validity and when there were multiple inflection points, the highest value was reported.

#### Activity assay

96-deep well plates were loaded with 3.5 mg of either amorphous PET film (Goodfellow ES30-FM-000145) or crystalline PET powder (Goodfellow ES30-PD-006031). Buffer concentration was 50 mM and the buffer composition varied by pH: sodium citrate for pH 4.5 and 5.5, sodium phosphate for pH 6.5 and 7.5, or glycine for pH 8.5. All buffers were prepared at room temperature. Each reaction had a 500 μL final volume with 5 μg of enzyme added. A single enzyme stock was used in one or two wells per condition (based on yield). The plates were heat-sealed to prevent evaporation and incubated at 50 or 70 °C without shaking for 48 h. For UV-Vis analysis, the assays containing crystalline powder were first filtered to remove any PET particles. For the filtered crystalline powder samples and amorphous film samples, 100 μL aliquots were evaluated for absorbance at 260 and 280 nm on a BioTek Synergy H1, and the results were blanked against no-enzyme controls. An average of TPA and MHET extinction coefficients (TPA: *ε*_260_ =  4.588 mM^−1^cm^−1^, *ε*_280_ = 1.300 mM^−1^cm^−1^ and MHET: *ε*_260_ = 6.519 mM^−1^cm^−1^, *ε*_280_ = 1.767 mM^−1^cm^−1^) was used to calculate product concentrations.

#### Validation set

We observed variation in the absolute activity values between experimental rounds performed on different dates, which may have arisen from differences in experimental factors such as protein purity and assay conditions such as buffer and substrate batch. To control for these factors as well as achieve full datasets with the enzymes tested in all conditions, we re-expressed, purified, and assayed the top designs alongside their parent enzymes in the same experimental run. This Validation Set included 72 EVdesigns, 10 ICCG-addition designs, *Tf*Cut2, and *Tf*Cut2-ICCG. For designs with initially low expression, we combined material from multiple purification wells to obtain sufficient protein (~300 μg per design) for full activity profiling across 16 assay conditions in duplicate. Using these approaches, we generated complete activity datasets for 81 of the 82 EVdesign enzymes.

### Crystallization, data collection and refinement

*E. coli* C41(DE3) cells carrying plasmids encoding *Tf*Cut2_EV2 and *Tf*Cut2_EV3 were grown in YT medium supplemented with 100 μg/mL kanamycin at 37 °C with shaking. Cultures were induced with 1 mM isopropyl *β*-D-1-thiogalactopyranoside (IPTG) upon reaching an OD600 of 0.4–0.7, then shifted to 18 °C and incubated for 16 h. Cells were harvested by centrifugation and stored at −80 °C until use. Cell pellets were resuspended in lysis buffer (50 mM Tris-HCl, 300 mM NaCl, pH 8.0) containing 0.1 mg/mL DNase I and 1 mg/mL lysozyme, and lysed by sonication (1 s on/1 s off; 4 min total “on” time). The lysate was clarified by centrifugation at 14,000 × *g* for 30 min at 4 °C, and the supernatant was filtered and applied to a pre-packed Ni-NTA column on an ÄKTA FPLC system equilibrated in 20 mM Tris-HCl, 300 mM NaCl, 20 mM imidazole, pH 8.0. After washing with 5 column volumes of equilibration buffer, proteins were eluted using a linear imidazole gradient up to 500 mM. Fractions containing *Tf*Cut2_EV2 or *Tf*Cut2_EV3 were pooled and dialyzed overnight at 4 °C into storage buffer (20 mM Tris-HCl, 300 mM NaCl, pH 8.0). Final protein concentrations were determined by absorbance at 280 nm using calculated extinction coefficients 37,150 (*Tf*Cut2_EV2) and 37,150 (*Tf*Cut2_EV3) M^-1^ cm^-1^.

For *Tf*Cut2_EV2, the purified protein was concentrated to 15 mg/mL and screened with 500 crystallization conditions with varying concentrations of the protein. The best crystals were obtained from SG1 screen well G1 (0.2 M Sodium acetate trihydrate, 0.1 M MES (pH 6.0), 20% PEG8K). Crystals were grown in sitting drops at 16 °C using a 1:0.9 ratio of protein to well solution and took around 3 weeks to grow. Crystals were transferred to a well solution supplemented with 20% glycerol and cryocooled in liquid nitrogen. Diffraction data were collected at the SSRL BL12-2 beamline using Dectris PILATUS EIGER 2XE 16M PAD detector. The crystals belonged to space group P1 with dimensions *a*=42.37 Å, *b*=42.38 Å, *c*=69.18 Å, *α* = 98.0°, *β* = 91.6°, *γ* = 101.9° with 2 monomers in the asymmetric unit. The data were processed with XDS^52^. The structure was solved by molecular replacement using AmoRE^53^ by using the model generated from a cutinase (PDB code: 5LUK)^54^ as the search model. Multiple rounds of manual model building using COOT^55^ and building using BUCCANEER^56^ were needed to successfully complete the model. The initial refinements were performed by REFMAC^57^ and the final rounds with PHENIX^58^. Final representative electron density maps are shown in Supplementary Fig. S12. The details of data collection and refinement are given in Supplementary Table S2.

For *Tf*Cut2_EV3, the purified protein was concentrated to around 12 mg/mL and screened with around 400 crystallization conditions. The crystals used for the data collection were from BCS screen well F10 (0.2 M AmSO_4_, 0.1 M Tris (pH 8.0) and 20% PEG smear broad). These crystals were difficult to grow and needed fine tuning attempts with protein concentration and drop ratio variations. Crystals were grown by sitting drop at 16 °C using a 1:0.9 ratio of protein to well solution and took around 6 weeks to appear. Crystals were transferred to a well solution supplemented with 15% glycerol and cryocooled in liquid nitrogen. Diffraction data were collected at the SSRL BL12-2 beamline using Dectris PILATUS EIGER 2XE 16M PAD detector. The crystals belonged to space group P6_1_ with dimensions *a* = 193.60 Å, *b* = 193.60 Å, *c* = 52.82 Å, *α* = 90°, *β* = 90°, *γ* = 120°. There were 3 monomers in the asymmetric unit. The data were processed with XDS^52^. The structure was solved by molecular replacement using AmoRE^53^ by using the *Tf*Cut2_EV2 structure as the search model. The completion of the structure required multiple cycles of manual building using the COOT^55^ program and multiple rounds of refinement with REFMAC^57^. The details of data collection and refinement are provided in Supplementary Table S2.

### Software used for data collection

The following software was used for data collection: Agilent BioTek Gen5 3.12 for analysis of absorbance assays (activity and BCA protein concentration), Bio-Rad CFX Manager 2.1 (software to import data and export to excel), CFX Maestro foranalysis of differential scanning fluorimetry (DSF, melting temperature determination), XDS (BUILT=20230630) to process the diffraction data.

### Software used for data analysis

The following software was used for data analysis: python 3.14.0, numba 0.63.0b1, pandas 2.3.3, evcouplings 0.2.1, biopython 1.86, seaborn 0.13.2, openpyxl 3.1.5, igraph 1.0.0, scikit-learn 1.7.2, jupyter 1.1.1, numpy 2.3.5, PyMOL 2.5.2, AMoRe CCP4 v9.0, COOT 0.9.8.95 EL, BUCCANEER CCP4 v9.0, REFMAC 5.8.0430, AlphaFold 3.

### Reporting summary

Further information on research design is available in the Nature Portfolio Reporting Summary linked to this article.

## Supplementary information

New Supplementary Information file

Supplementary Data File 1

Reporting Summary

Transparent Peer Review file

## Source data

Source Data

## Data Availability

There are no restrictions on data access. Source Data are provided with this paper, which includes all the data used in main and Supplementary figures. All pre-processed data are available in Supplementary Data File1 provided by the journal. All code, raw and processed data are accessible at https://github.com/gauthierscience/petase-protein-design^59^. The output of EVcouplings runs, including the parameters used as well as the MSAs, models, and output metrics are available on Zenodo^51^. The atomic coordinates and structure factors have been deposited in the Protein Data Bank for *Tf*Cut2_EV2 (PDB ID: 9PM1) and *Tf*Cut2_EV3 (PDB ID: 9PM2). The 84 Validation Set plasmids that were generated in this study are available from AddGene (accession codes 257140 - 257223). Source data are provided with this paper.

## Code availability

All data and custom analysis code are available at: https://github.com/gauthierscience/petase-protein-design^59^.
