# Supplementary material for: Iterative computational and rational design generates diverse PET hydrolases for plastic depolymerization
Source: Nat Commun. 2026 Nov 3;17:11371. doi: 10.1038/s41467-026-78471-3 (PMC13731650; doi:10.1038/s41467-026-78471-3)
Supplement: Supplementary file 1 [file 41467_2026_78471_MOESM1_ESM.pdf]

Supplementary Information

Iterative computational and rational design generates diverse PET hydrolases for plastic depolymerization

Brenna Norton-Baker[1,2,3], Benjamin Fram[4,5], Nicole N. Thadani[4,6], Evan Komp[1,3], Yang Su[4], Olateju O. Storment[1,2], Natasha P. Murphy[1,2,3], Samuel Lim[4,7], Erika Erickson[1,2], Nathan Rollins[4], Japheth E. Gado[1,2], Courtney A. Shearer[4], Irimpan I. Mathews[8], Ian Truebridge[9,10], Maria Llases[9], Christopher D. Bahl[9,11,12], Ritimukta Sarangi[8], Debora S. Marks[4], John E. McGeehan[1,2], Chris Sander[4,5], Gregg T. Beckham[1,2,3,✉], and Nicholas P. Gauthier[4,5,✉]

[1]Renewable Resources and Enabling Sciences Center, National Laboratory of the Rockies, Golden, Colorado 80401, United States
[2]BOTTLE Consortium, Golden, Colorado 80401, United States
[3]Agile BioFoundry, Emeryville, California 94608, United States
[4]Department of Systems Biology, Harvard Medical School, Boston, Massachusetts 02115, United States
[5]Department of Data Sciences, Dana-Farber Cancer Institute, Boston, Massachusetts 02115, United States
[6]Present address: Apriori Bio, Somerville, MA, United States
[7]Integrated Science and Engineering Division, Underwood International College, Yonsei University, Incheon 21983, South Korea
[8]SLAC National Accelerator Laboratory, Stanford Synchrotron Radiation Lightsource, Menlo Park, California 94025, United States
[9]Institute for Protein Innovation, Boston, MA, United States
[10]Present address: Aera Therapeutics, Cambridge, MA, United States
[11]Present address: AI Proteins, Boston, MA, United States
[12]Dana-Farber/Boston Children's Cancer and Blood Disorders Center; Harvard Medical School, Boston, MA, United States
[✉]Correspondence should be addressed to G.T.B. (gregg.beckham@nlr.gov) and N.P.G. (nicholas.gauthier.research@gmail.com)

Contents

Supplementary Note . . . 3
Supplementary Figures . . . 4
Supplementary Tables . . . 16
References . . . 18

List of Figures

S1 Predicted fitness of designs from eight distinct scaffolds . . . 4
S2 Raw activity of designs based on seven distinct scaffolds . . . 5
S3 Comparison of the *Tf*Cut2 variant used as a wild-type control in the scaffold data set with wild-type *Tf*Cut2 . . . 6
S4 Raw activity of designs from the diversity set . . . 7
S5 Performance comparison of designs from the diversity set across experimental conditions . 8
S6 Structural alignment of *Tf*Cut2 and LCC-ICCG . . . 9
S7 Raw activity of designs based on *Tf*Cut2-ICCG . . . 10
S8 The effect of mutation count on performance across conditions of designs based on the *Tf*Cut2-ICCG scaffold . . . 11
S9 The effect of mutation count on activity at 50 °C and 70 °C of designs based on the *Tf*Cut2-ICCG scaffold . . . 12
S10 Raw activity of top designs and their parental ancestry . . . 13
S11 Structural comparison of *Tf*Cut2 with X-ray structures of two *Tf*Cut2-based designs . . . 14
S12 Representative electron density maps of two *Tf*Cut2-based designs . . . 15

List of Tables

S1 Sequence and structural similarity between PETase scaffolds . . . 16
S2 Crystallographic parameters, data collection and refinement statistics . . . 17

Supplementary Note

Supplementary Note S1. Additional sequence designs. In addition to the standard evolution-informed design pipeline (see Methods) we also generated and tested a number of designs that acted either as internal controls or were explorations into alterations to the design methodology. For clarity we do not provide a lengthy discussion / analysis of these sequences in the main text, but we have included these sequences and their cognate expression, melting temperatures, and enzymatic activity measurements in **Supplementary Data 1** as an open community resource.

Although the focus of the main text was on *Tf*Cut2, we did explore additional evolution-informed designs from several scaffolds throughout the project. These included iterative designs on top of 611 and 611-ICCG, a small number of HotPETase designs (n=9), and a large number of LCC-ICCG designs (n=29) that contained >70 mutations. Data for all of these designs have also been deposited in the **Supplementary Data 1** file.

Supplementary Figures

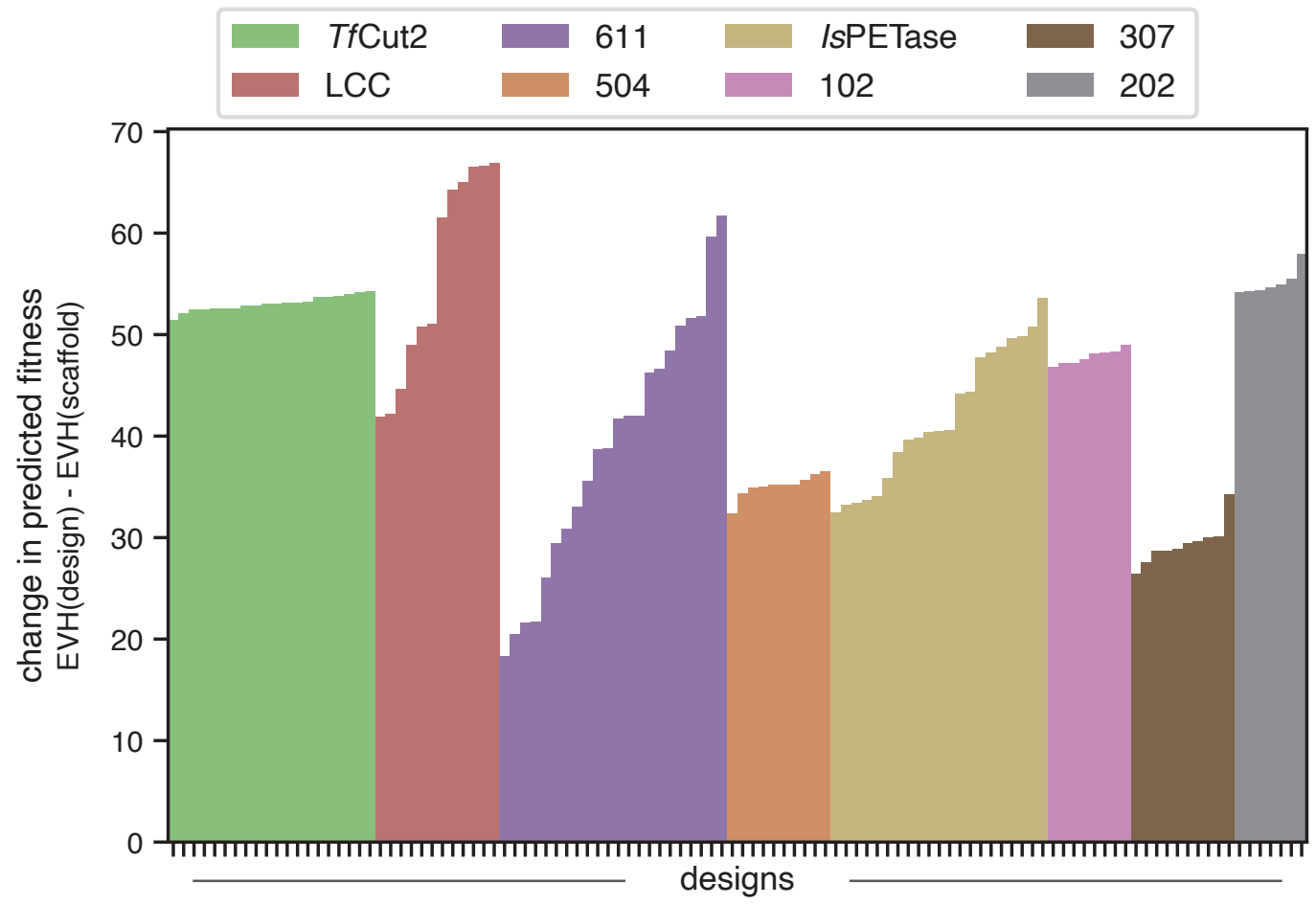

Supplementary Figure S1. All designs tested during scaffold evaluation had increases in predicted fitness. Y-axis indicates the change in predicted fitness defined as the evolutionary hamiltonian (EVH) of the design minus the EVH of the scaffold sequence. Values are first sorted by scaffold and then the change in predicted fitness. Each tick on the X-axis is a single design. Colors indicate the design scaffold. Source data are provided in the Source Data file.

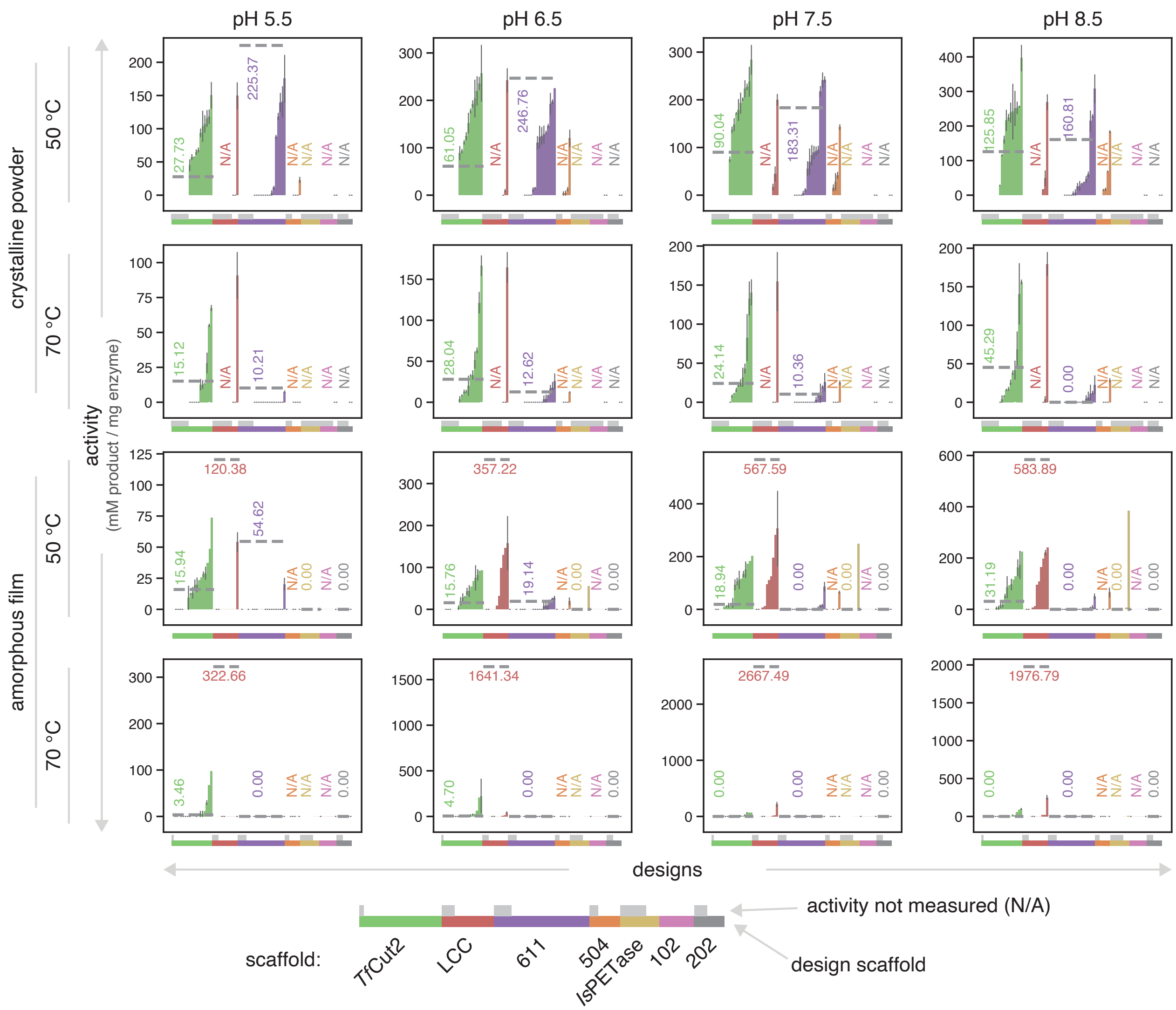

Supplementary Figure S2. Raw activity of designs based on seven distinct scaffolds. Each of the 16 plots shows enzymatic activity in a specific condition (specific substrate, temperature, pH). Within each plot, designs are ordered first by the scaffold from which they were derived, and then by their average activity within that condition with N/A values being sorted first (before zero). **Bar colors** indicate design scaffold: green=*Tf*Cut2, red=LCC, purple=611, orange=504, yellow=*Is*PETase, pink=102, gray=202. **Dashed lines and numerical labels**: the activity of the scaffold. Absence of the dashed line and an **N/A** label indicate that the scaffold was not tested due to low purification yield. **Gray bars under x-axes**: show designs that were not tested (i.e., N/A). **Colored bars under x-axes**: define scaffold x-limits (colors as above). **Range Bars**: extend from the minimum and maximum values of two replicates. Samples without range bars had low protein yield, and height represents activity for a single replicate. Source data are provided in the Source Data file.

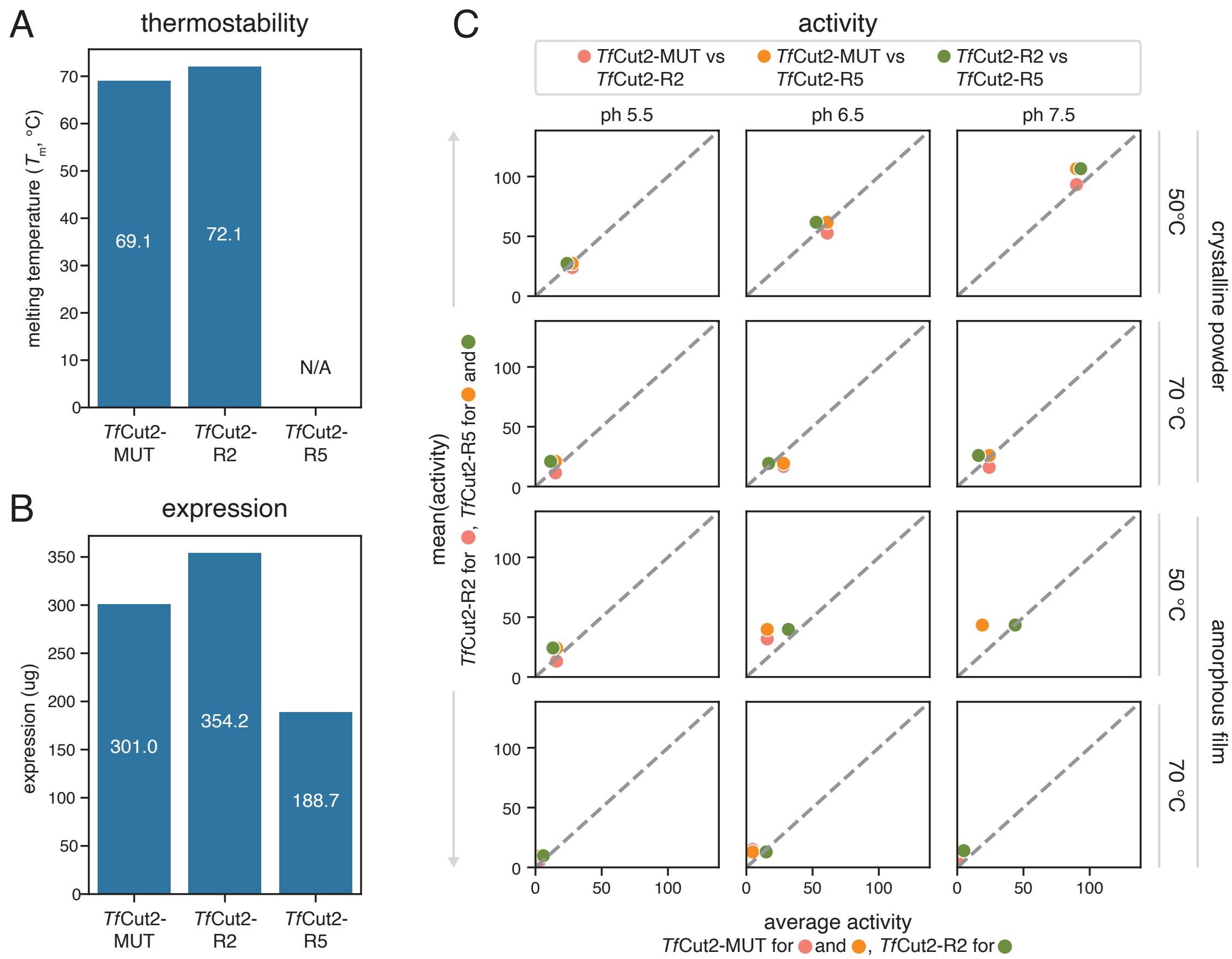

Supplementary Figure S3. The *Tf*Cut2 variant (*Tf*Cut2-MUT) used as a wild-type control in the initial scaffold-selection experiments had similar thermostability, expression, and activity as wild-type *Tf*Cut2. Due to an input error, the "wild-type *Tf*Cut2" variant (*Tf*Cut2-MUT) characterized in the scaffold-selection round contained an additional amino acid (tyrosine) on the C-terminus of the protein, which could affect our interpretation of design performance if it behaved differently than wild-type *Tf*Cut2. *Tf*Cut2-R2: wild-type *Tf*Cut2 characterized in the diversity round. *Tf*Cut2-R5: wild-type *Tf*Cut2 characterized the final iterative design and parent round. **(A)** Thermostability of the *Tf*Cut2-MUT compared to *Tf*Cut2-R2. Thermostability was not characterized for *Tf*Cut2-R5. **(B)** Expression yield per well of a 24-well plate. **(C)** Average activity measurements in all shared conditions (2 substrates, 2 temperatures, 3 pHs). **Pink dot**: comparison of *Tf*Cut2-MUT (x-axis) to *Tf*Cut2-R2 (y-axis). **Orange dot**: comparison of *Tf*Cut2-MUT (x-axis) to *Tf*Cut2-R5 (y-axis). **Green dot**: comparison of *Tf*Cut2-R2 (x-axis) to *Tf*Cut2-R5 (y-axis). Activity units = mM product / mg enzyme. Source data are provided in the Source Data file.

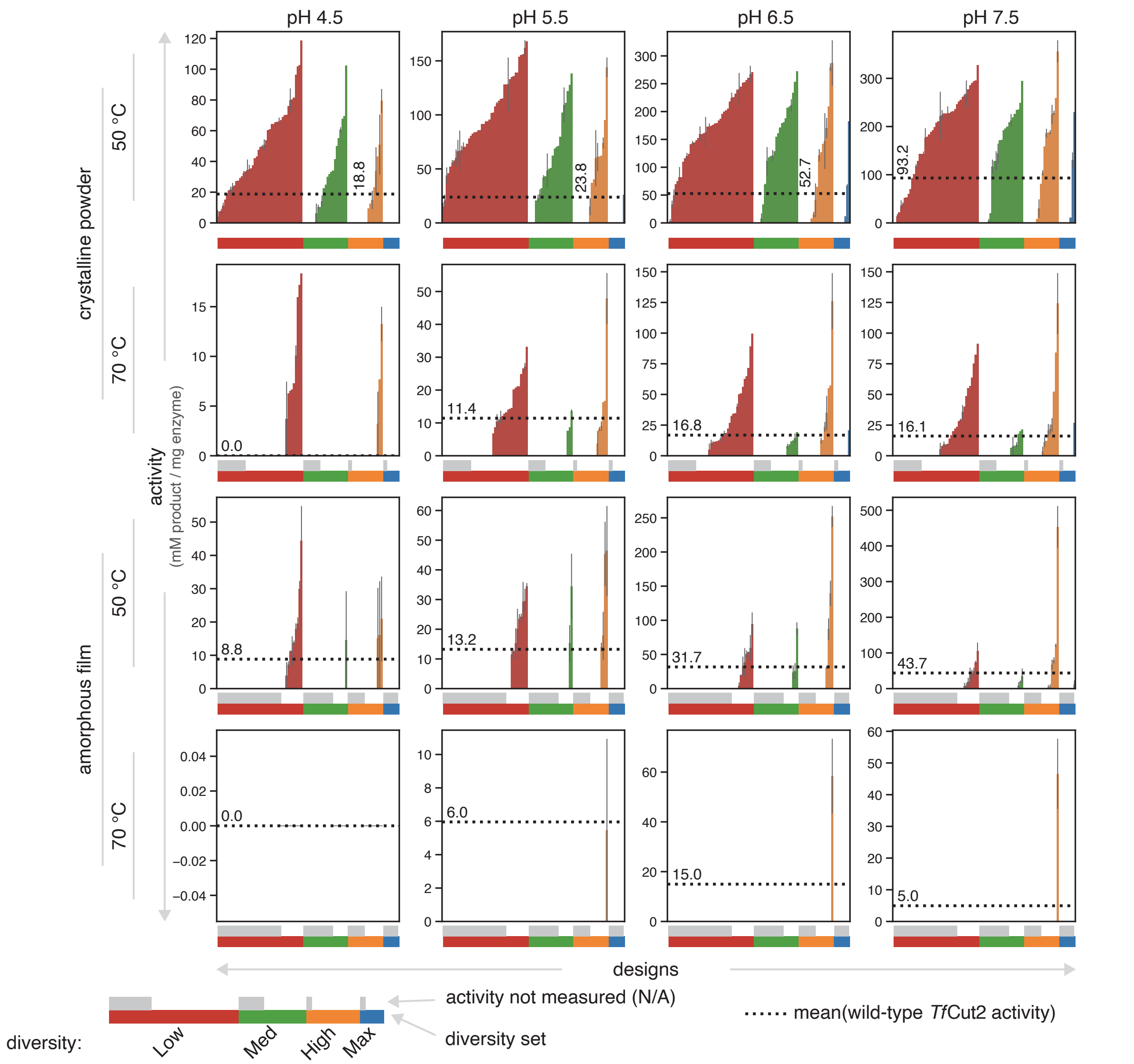

Supplementary Figure S4. Waterfall plot of all activity data for the diversity-set designs shows many have higher activity than wild-type *Tf*Cut2. Each of the 16 plots show enzymatic activity in a specific condition (substrate, temperature, pH). Within each plot, designs are ordered first by diversity (**Low, Med, High, Max**), and then by average activity within that condition with N/A values being sorted first (before zero). Each colored bar depicts the mean activity (one or two replicates depending on protein yield) of a single design in the specific condition. **Bar colors**: Red = **Low** diversity set, Green = **Med** diversity set, Yellow = **High** diversity set, and Blue = **Max** diversity set. **Black dashed line with value**: activity of the control *Tf*Cut2 enzyme (mean of two replicates). **Gray bars under x-axes**: show designs that were not tested (i.e., N/A). **Colored bars under x-axes**: define diversity set x-limits (colors as above). **Range Bars**: extend from the minimum and maximum values of two replicates. Samples without range bars had low protein yield, and height represents activity for a single replicate. Source data are provided in the Source Data file.

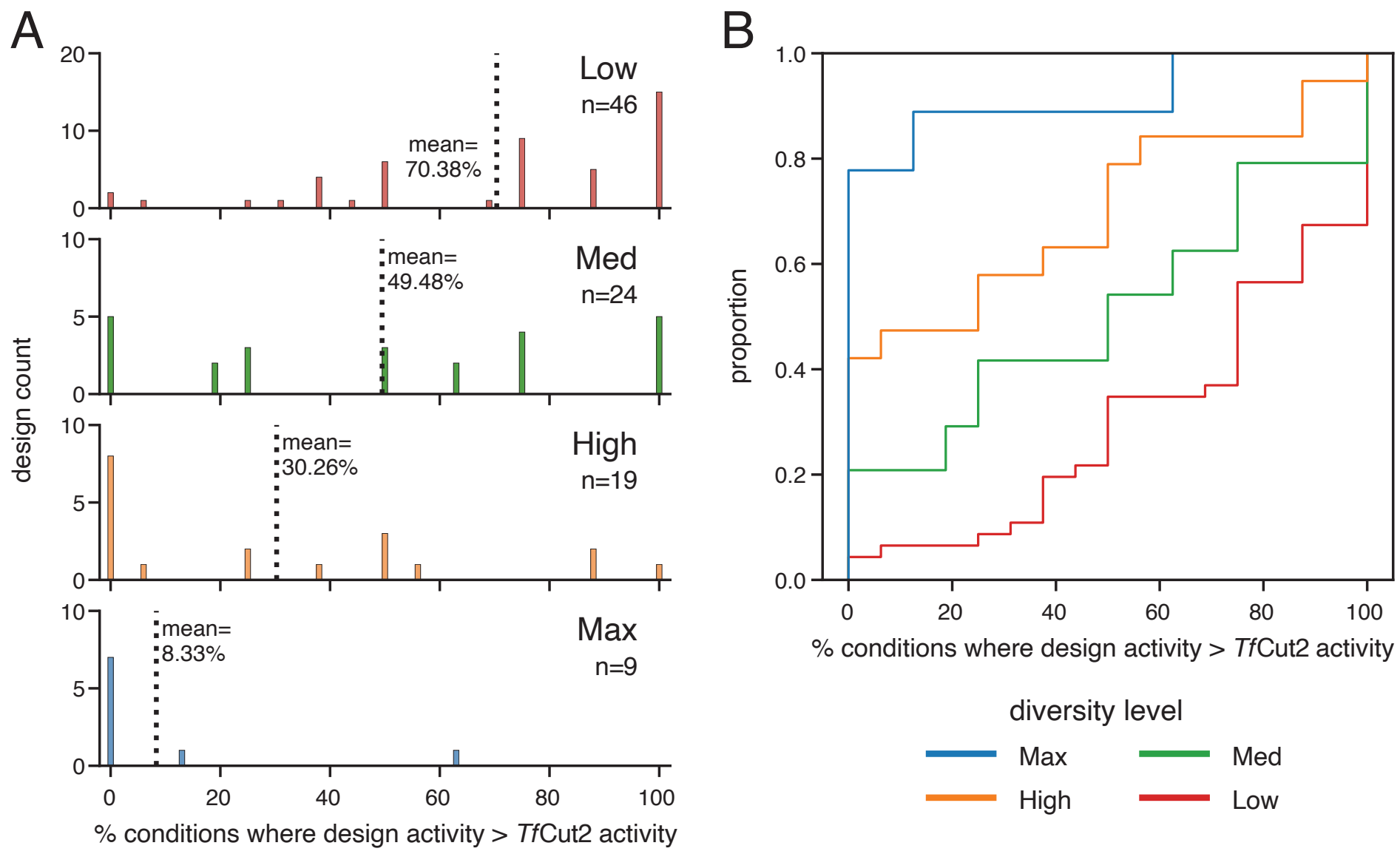

Supplementary Figure S5. As design set diversity decreases, on average designs outperform wild-type *Tf*Cut2 in more conditions. Activity was determined for all *Tf*Cut2-based designs that had sufficient protein yield (n=46, 24, 19, and 9 for the **Low**, **Med**, **High**, and **Max** diversity sets, respectively). The percentage better than *Tf*Cut2 is calculated for each design as: the number of conditions in which the average activity was better than wild-type *Tf*Cut2 divided by the number of conditions the design was tested in (i.e., 16 conditions for high yield designs, and 8 or 4 conditions for low expressing designs) multiplied by 100 to give a percentage. **(A)** Histogram of all tested designs with bin width equal to one. Designs for the **Low**, **Med**, **High**, and **Max** diversity sets are shown in descending order. **(B)** Cumulative distribution function (CDF) representation of the same data in (A). Source data are provided in the Source Data file.

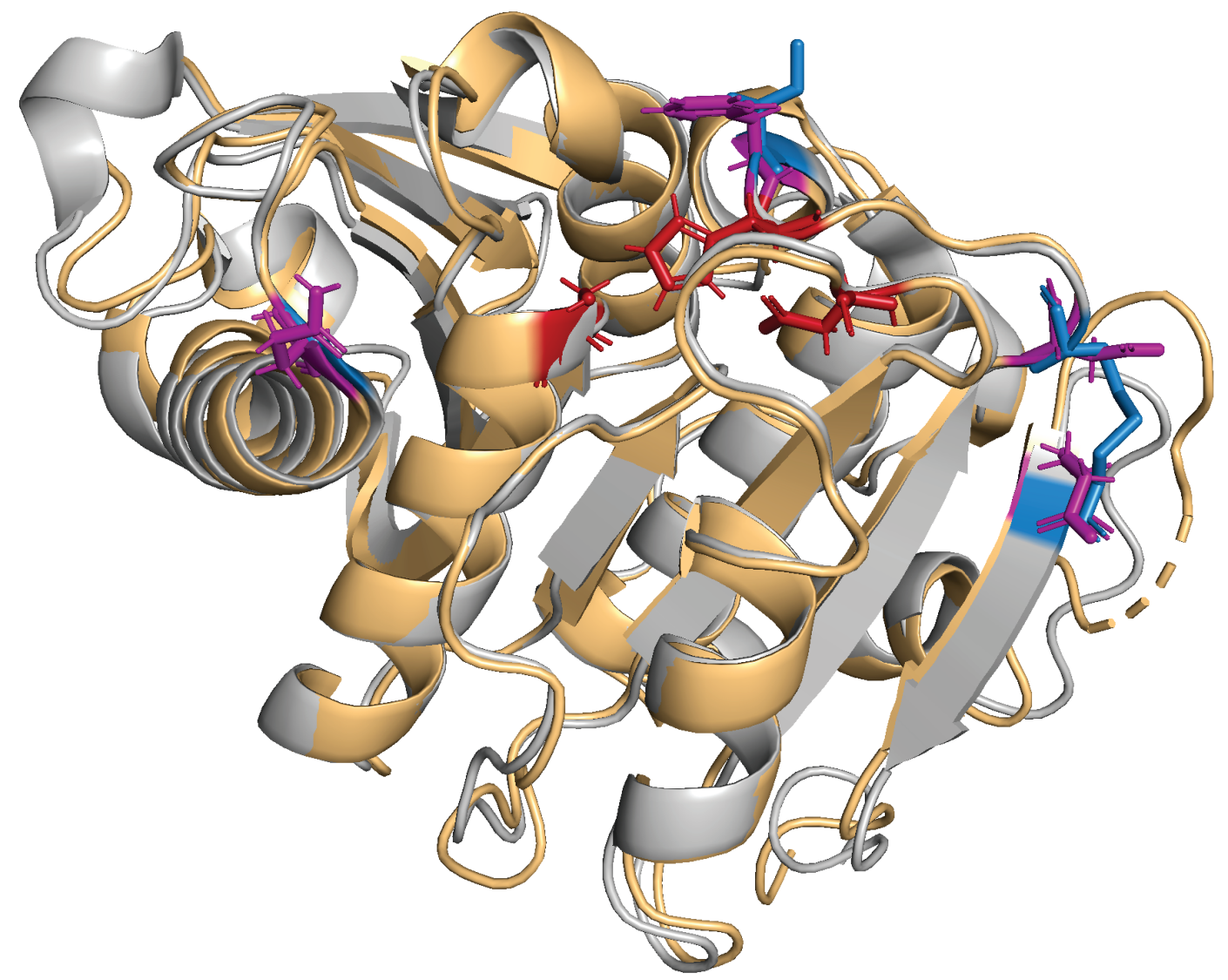

Supplementary Figure S6. Structural alignment of published X-ray structures of *Tf* Cut2 (light orange, PDB: chain A of 4CG1)[1] and LCC-ICCG (gray, PDB: chain A of 7VVC)[2]. Alignment, which was performed by `align` command in PyMOL, shows nearly identical backbone conformations between the two structures (RMSD of 0.551 for 1379 aligned atoms). The ICCG residues of LCC-ICCG (243I, 238C, 283C, 127G) are shown in stick representation in blue. The residues in *Tf* Cut2 that structurally align with the ICCG residues are shown in stick representation in purple (F209I, D204C, E253C, Q92G). The catalytic residues in *Tf* Cut2 (130S, 176D, 208H) are shown in stick representation in red.

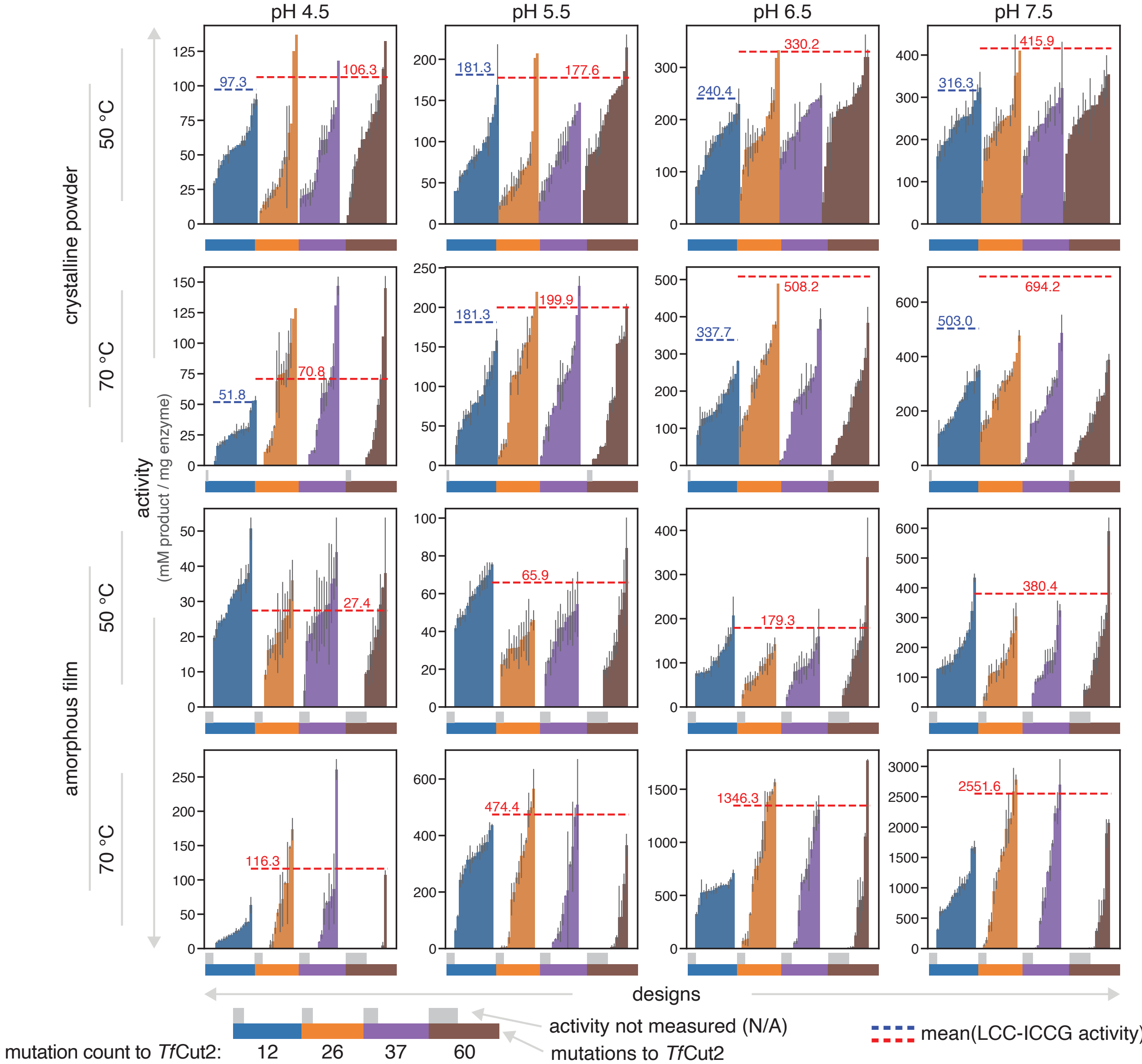

Supplementary Figure S7. Raw activity of *Tf*Cut2-ICCG-based designs shows all are active in at least one condition and most are active in many conditions. Each of the 16 plots show enzymatic activity in a specific condition (substrate, temperature, pH). All designs based on *Tf*Cut2-ICCG with sufficient purification yield (n=74 of 80) are included. Each colored bar depicts the mean activity (two replicates) of a single design in the specific condition. **Bar colors**: blue, orange, purple, and brown designs with 12, 26, 37, and 60 mutations, respectively. Designs with 26, 37, and 60 mutations were tested in one experimental round, while designs with 12 mutations were tested in a separate round. As we observed variation between rounds, we refrain from comparison of the absolute activity values between individual rounds. **Dashed lines and value**: average activity of the control LCC-ICCG enzyme (two replicates, expressed in plate with designs) tested with designs that contained 12 mutations (blue dashed line) or 26, 37, and 60 mutations (red dashed line). The blue dashed line is not included on amorphous film as the LCC-ICCG enzyme yield was too low in that experimental round. **Range Bars**: extend from the minimum and maximum values of two replicates. Source data are provided in the Source Data file.

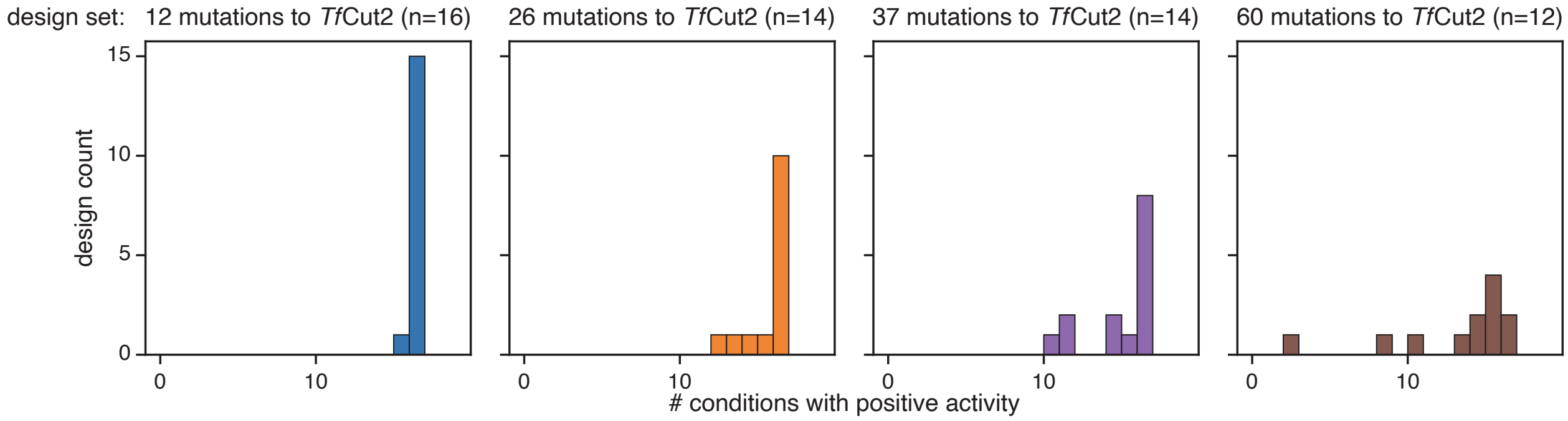

Supplementary Figure S8. The number of conditions in which *Tf* Cut2-ICCG-based designs are active decreases with increasing mutation count. Each bar represents the number of designs (y-axis) and the number of conditions in which they had positive activity (x-axis). In order to focus on activity rather than other variables (e.g., expression differences), only designs tested in all 16 conditions (2 substrates × 2 temperatures × 4 pHs) are included. Source data are provided in the Source Data file.

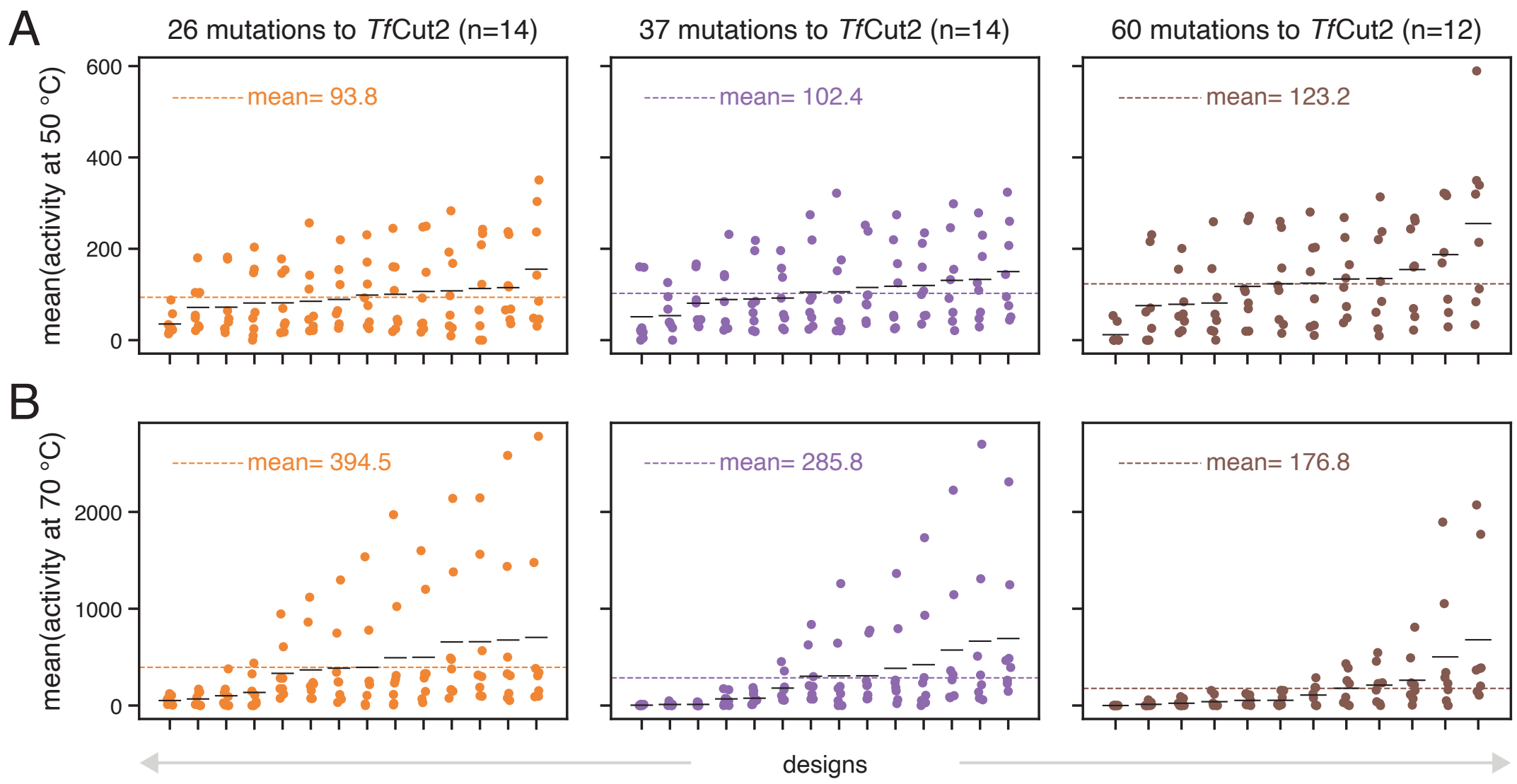

Supplementary Figure S9. Increased mutational burden in designs based on *Tf*Cut2-ICCG is associated with higher average activity at 50 °C and lower average activity at 70 °C. In order to focus on activity rather than other variables (e.g., expression differences), only designs that were tested in all 16 conditions (2 substrates x 2 temperatures x 4 pHs) are included. **(A)** The average activity (two replicates, y-axis) in the 50 °C and **(B)** 70 °C conditions are depicted as eight individual dots for each design (column on x-axis). Activity units = mM product / mg enzyme. The eight conditions tested at 50 °C and 70 °C were two substrates (amorphous film and crystalline powder) in four buffers (pH 4.5, pH 5.5, pH 6.5, and pH 7.5). Columns in each plot: individual *Tf*Cut2-ICCG-based designs. **Dots**: the mean of the design activity (2 replicates) for a single particular condition. **Black line**: mean of all eight conditions for that design. Designs that contain 26, 37, and 60 mutations are shown in the plots on the left (orange dots), middle (purple dots), and right (brown dots), respectively. As absolute activity measurements varied between rounds, we included only designs tested in the same experimental round and therefore omitted *Tf*Cut2-ICCG designs with 12 mutations (blue in main text). Source data are provided in the Source Data file.

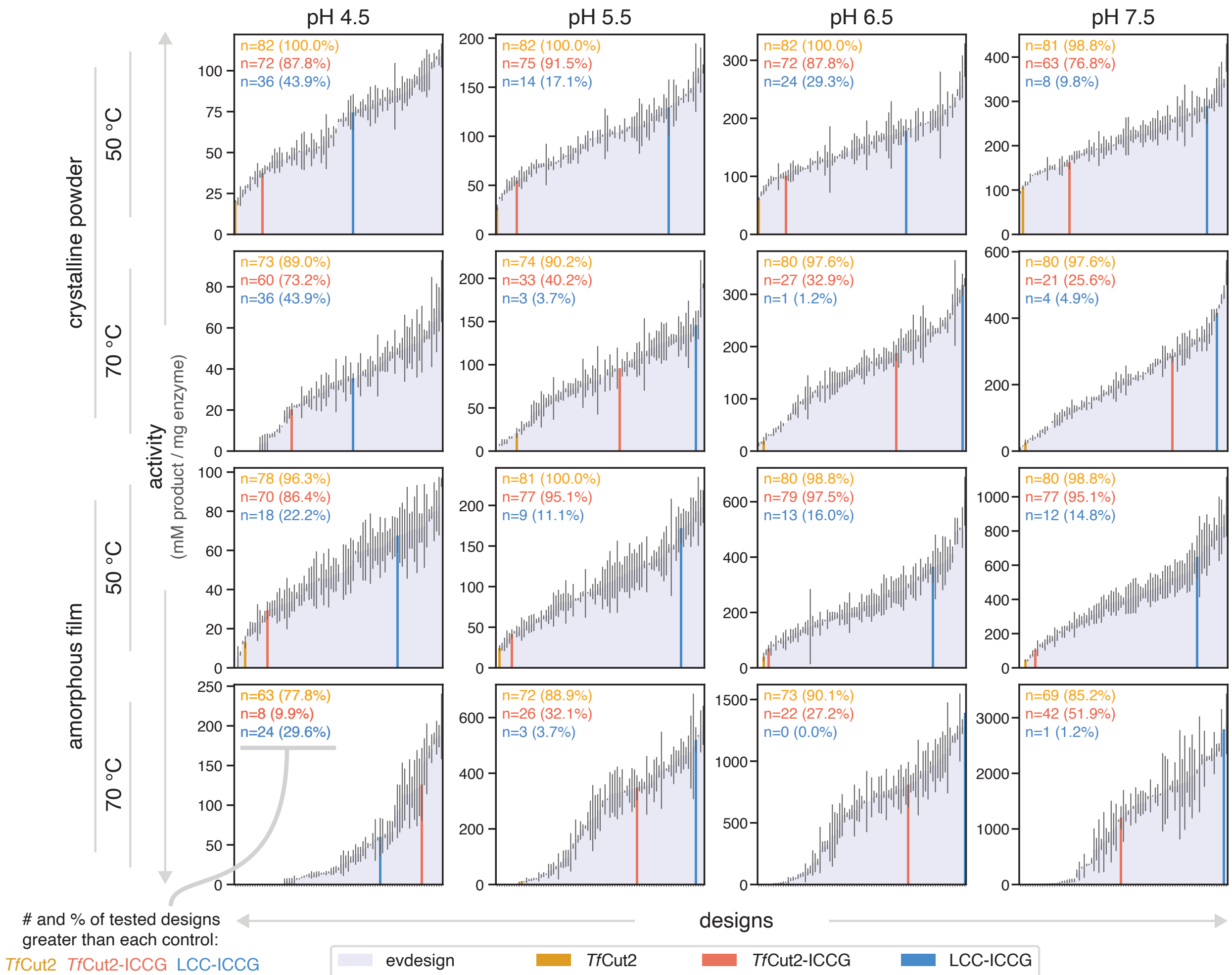

Supplementary Figure S10. Raw activity of top designs, together with all of their parental ancestry, from a single experimental round. Each of the 16 plots show enzymatic activity in a specific condition (substrate, temperature, pH). Each bar depicts the mean activity (two replicates) of a single design in the specific condition. Controls: *Tf*Cut2 is orange, *Tf*Cut2-ICCG is red, and LCC-ICCG is blue. Text in the upper left corner of each plot indicates the number and percentage of designs for which the mean activity is higher than the mean activity of each control (colored by control). A single design had low expression and was untested in all eight amorphous film conditions. All other designs and controls are depicted in all 16 conditions. **Range Bars**: extend from the minimum and maximum values of two replicates. Source data are provided in the Source Data file.

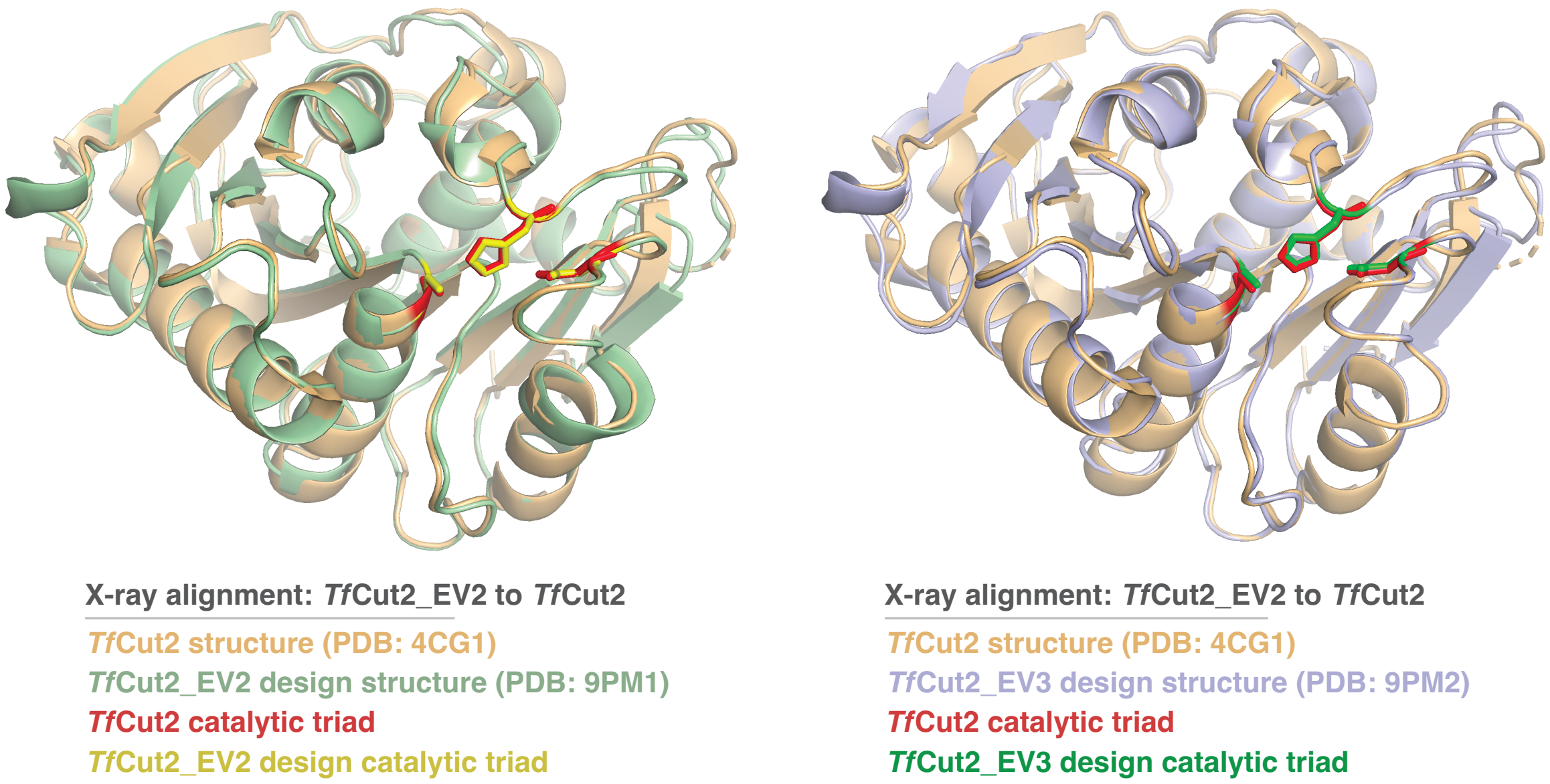

Supplementary Figure S11. Structural comparison of the published X-ray structure of *Tf*Cut2 (light orange, PDB: chain A of 4CG1)[1] with X-ray structures from this study, *Tf*Cut2_EV2 (left figure in green, PDB: 9PM1) and *Tf*Cut2_EV3 (right figure in purple, PDB: 9PM2). Alignments were performed with the `align` command in PyMOL. The corresponding active site triad residues (130S, 176D, 208H) are shown in stick representation in red for *Tf*Cut2, yellow for *Tf*Cut2_EV2, and green for *Tf*Cut2_EV3.

A Representative electron density map (stereo image) of design *Tf*Cut2_EV2 (PDB: 9PM1)

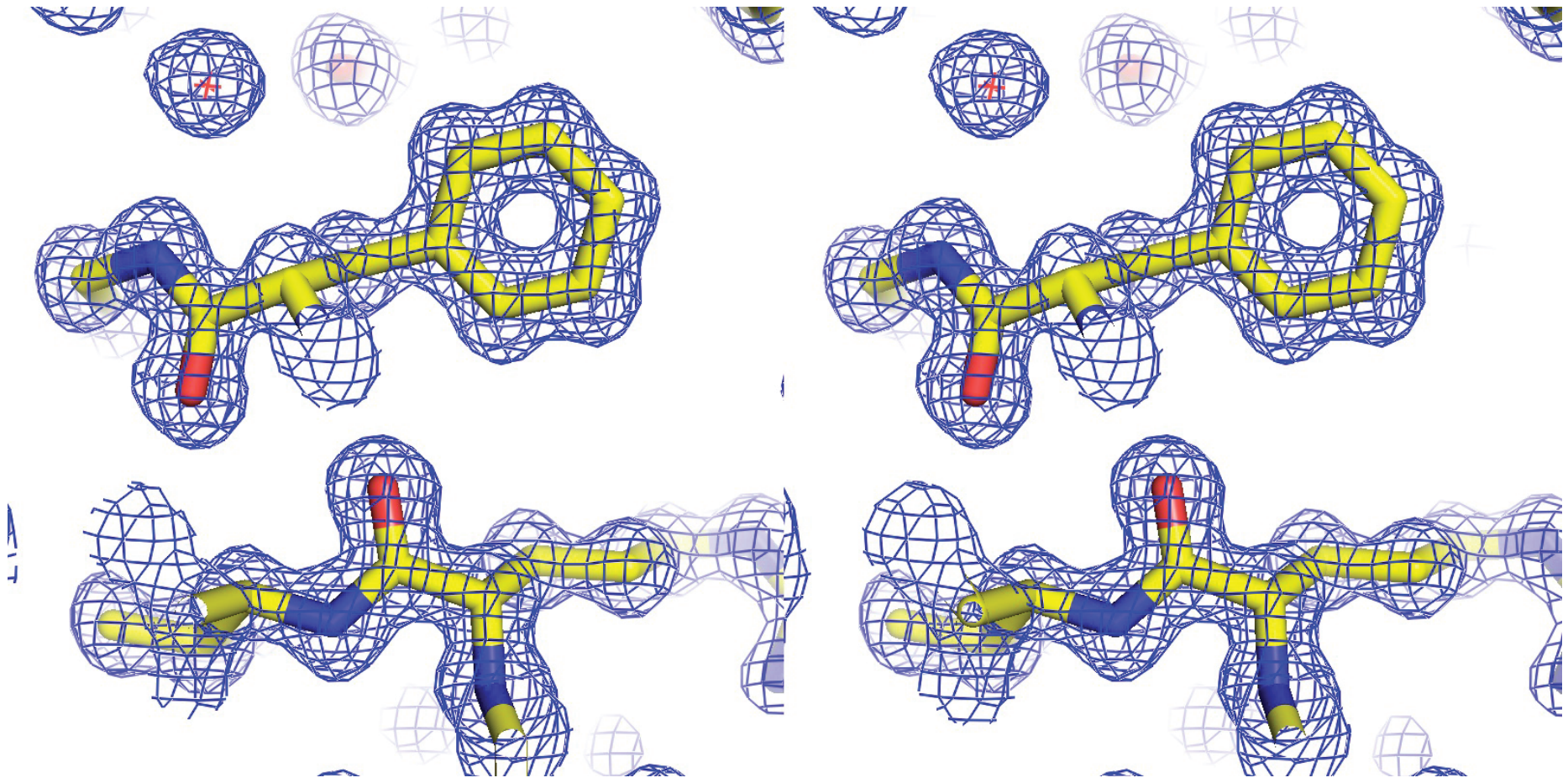

B Representative electron density map (stereo image) of design *Tf*Cut2_EV3 (PDB: 9PM2)

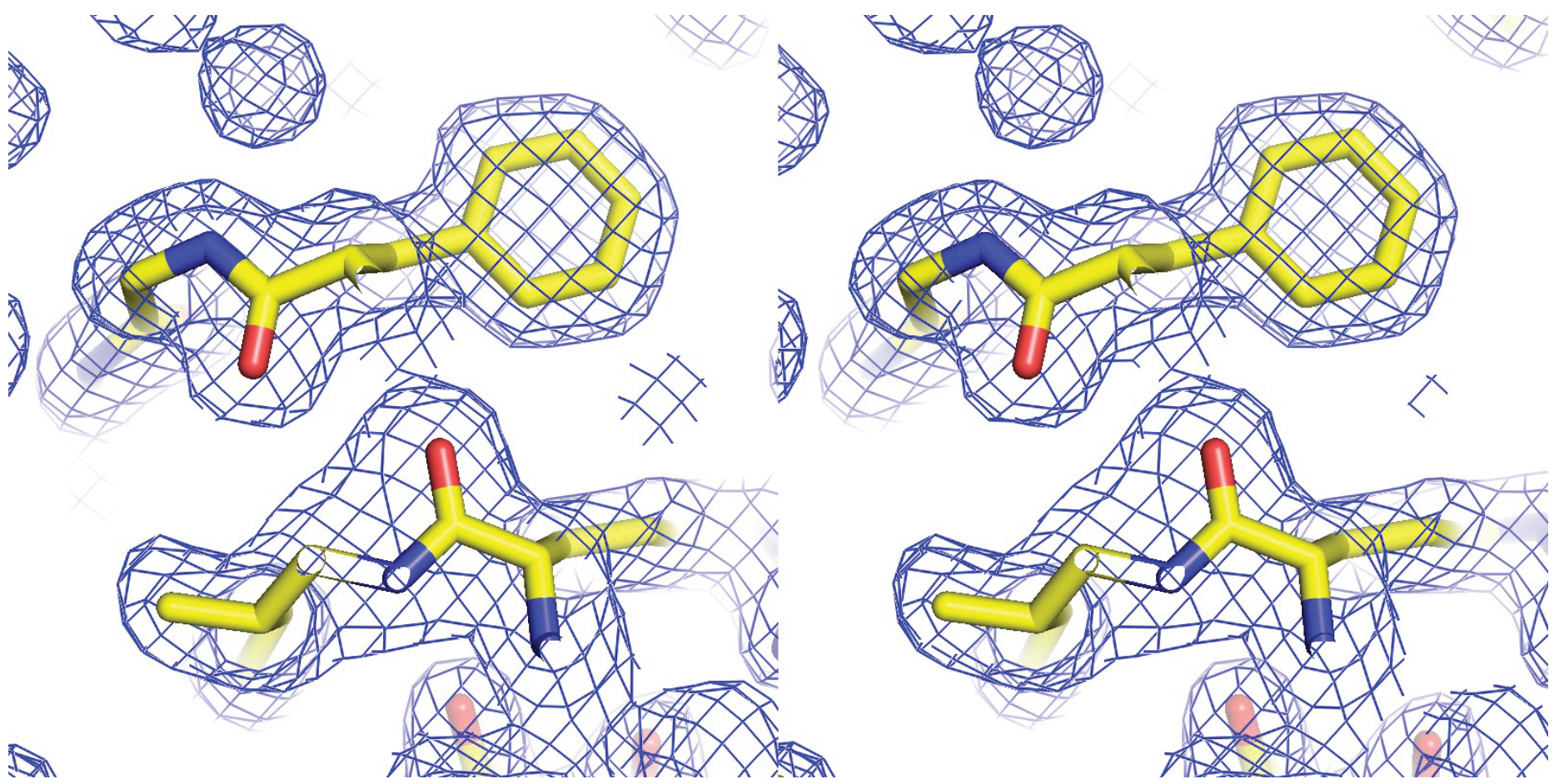

Supplementary Figure S12. Representative electron density maps of two *Tf*Cut2-based designs. **(A)** Stereo image showing representative $2F_o - F_c$ electron density of *Tf*Cut2_EV2 (PDB: 9PM1, resolution 1.1 Å) centered around Phe51 contoured at 0.5 e/A^3. **(B)** Stereo image showing representative $2F_o - F_c$ electron density of *Tf*Cut2_EV3 (PDB: 9PM2, resolution 1.7 Å) centered around Phe51 contoured at 0.3 e/A^3.

Supplementary Tables

A Sequence identity between PETase scaffolds

	102	202	307	504	611	IsPETase	LCC	TfCut2
**102**	100.0%	10.4%	0.8%	7.1%	8.7%	9.6%	7.6%	8.6%
**202**		100.0%	13.4%	14.7%	16.5%	14.8%	16.1%	16.3%
**307**			100.0%	13.0%	12.4%	11.3%	13.3%	13.2%
**504**				100.0%	49.1%	52.1%	50.8%	49.4%
**611**					100.0%	45.2%	52.5%	60.5%
**IsPETase**						100.0%	46.6%	50.8%
**LCC**							100.0%	56.1%
**TfCut2**								100.0%

B RMSD between PETase scaffold structures

	102	202	307	504	611	IsPETase	LCC	TfCut2
**102**	0.00 Å	3.74 Å	5.37 Å	3.32 Å	3.37 Å	3.35 Å	3.48 Å	3.43 Å
**202**		0.00 Å	2.29 Å	2.28 Å	2.23 Å	2.29 Å	2.22 Å	1.95 Å
**307**			0.00 Å	2.42 Å	2.31 Å	2.17 Å	2.42 Å	2.34 Å
**504**				0.00 Å	1.10 Å	1.00 Å	1.19 Å	1.27 Å
**611**					0.00 Å	1.26 Å	1.10 Å	0.86 Å
**IsPETase**						0.00 Å	1.40 Å	1.35 Å
**LCC**							0.00 Å	1.04 Å
**TfCut2**								0.00 Å

C Coverage (# aligned residues / average sequence length)

	102	202	307	504	611	IsPETase	LCC	TfCut2
**102**	1.000	0.596	0.397	0.478	0.503	0.488	0.502	0.491
**202**		1.000	0.728	0.712	0.724	0.717	0.716	0.703
**307**			1.000	0.619	0.622	0.615	0.616	0.615
**504**				1.000	0.962	0.974	0.968	0.976
**611**					1.000	0.966	0.979	0.983
**IsPETase**						1.000	0.975	0.975
**LCC**							1.000	0.988
**TfCut2**								1.000

Supplementary Table S1. Sequence and structural similarity between PETase scaffolds. Pairwise TM-align alignments were conducted on AlphaFold 3 predicted structures of each protein. **(A)** Symmetric percent identity computed as the quantity of identical and equivalent residues in the structural alignment over the average sequence length. **(B)** Root Mean Square Deviation (RMSD) of equivalent residues from the structural alignment. **(C)** The coverage of the aligned models (# of aligned residues / average sequence length) that was used in the sequence identity and RMSD reported in **A** and **B**, respectively.

	*Tf*Cut2_EV2 (PDB ID: 9PM1)	*Tf*Cut2_EV3 (PDB ID: 9PM2)
**Crystallographic parameters**		
Space group	P1	$P6_1$
Unit-cell dimensions	42.37, 42.38, 69.18 Å<br>98°, 91.6°, 101.9°	193.60, 193.60, 52.82 Å<br>90°, 90°, 120°
**Data collection statistics**		
Resolution limits (outer shell) (Å)	37.7-1.08 (1.11-1.08)	38.5-1.70 (1.74-1.70)
Observed reflections (outer shell)	948,835 (35,864)	3,418,683 (242,925)
Unique reflections (outer shell)	178,134 (14,785)	123,744 (9,056)
Completeness (outer shell)	89.2 (69.7)	99.2 (99.1)
$CC_{1/2}$ (outer shell)	99.8 (81.4)	99.8 (60.6)
Redundancy	5.3	27.6
R_{sym} [a]	8.9 (81.5)	20.8 (281.3)
Mean $I/\sigma(I)$ (outer shell)	10.9 (3.5)	12.2 (1.6)
**Refinement statistics**		
Resolution limits (Å)	37.7-1.08	38.5-1.7
Number of reflections (%)	178,115 (89.1)	117,556 (99.2)
Reflections used for R_{free}	8,907	6,188
R_{factor} [b] (%)	13.6	14.3
R_{free} (%)	15.9	16.7
Model contents (average B(Å^2))		
Protein atoms	4,163 (11.9)	5,928 (25.6)
Ligand	0	0
Ion/buffer	13 (30.4)	72 (60.3)
Water molecules	694 (28.4)	944 (39.2)
RMS deviations		
Bond length (Å)	0.012	0.011
Bond angle (°)	1.27	1.86
Ramachandran (favored (%)/outliers)	98/0	98/0

Supplementary Table S2. Crystallographic parameters, data collection and refinement statistics for *Tf*Cut2_EV2 and *Tf*Cut2_EV3.

[a] $R_{sym} = \sum |I_{avg} - I_i|$

[b] $R_{factor} = \sum |F_p - F_{p,calc}| / \sum |F_p|$, where F_p and $F_{p,calc}$ are the observed and calculated structure factors; R_{free} is calculated with 5% of the data.

References

1. Roth, C. *et al.* Structural and functional studies on a thermostable polyethylene terephthalate degrading hydrolase from thermobifida fusca. *Appl. Microbiol. Biotechnol.* **98**, 7815–7823 (2014).

2. Zeng, W. *et al.* Substrate-binding mode of a thermophilic pet hydrolase and engineering the enzyme to enhance the hydrolytic efficacy. *ACS Catal.* **12**, 3033–3040 (2022).
