# Supplementary material for: Iterative computational and rational design generates diverse PET hydrolases for plastic depolymerization
Source: Nat Commun. 2026 Nov 3;17:11371. doi: 10.1038/s41467-026-78471-3 (PMC13731650; doi:10.1038/s41467-026-78471-3)
Supplement: Supplementary file 2 [file 41467_2026_78471_MOESM3_ESM.pdf]

nature portfolio

Corresponding author(s): Nicholas Gauthier and Gregg Beckham

Last updated by author(s): August 26, 2026

Reporting Summary

Nature Portfolio wishes to improve the reproducibility of the work that we publish. This form provides structure for consistency and transparency in reporting. For further information on Nature Portfolio policies, see our Editorial Policies and the Editorial Policy Checklist.

Statistics

For all statistical analyses, confirm that the following items are present in the figure legend, table legend, main text, or Methods section.

n/a	Confirmed	
[ ]	[x]	The exact sample size (*n*) for each experimental group/condition, given as a discrete number and unit of measurement
[ ]	[x]	A statement on whether measurements were taken from distinct samples or whether the same sample was measured repeatedly
[x]	[ ]	The statistical test(s) used AND whether they are one- or two-sided<br>*Only common tests should be described solely by name; describe more complex techniques in the Methods section.*
[x]	[ ]	A description of all covariates tested
[x]	[ ]	A description of any assumptions or corrections, such as tests of normality and adjustment for multiple comparisons
[ ]	[x]	A full description of the statistical parameters including central tendency (e.g. means) or other basic estimates (e.g. regression coefficient) AND variation (e.g. standard deviation) or associated estimates of uncertainty (e.g. confidence intervals)
[x]	[ ]	For null hypothesis testing, the test statistic (e.g. *F*, *t*, *r*) with confidence intervals, effect sizes, degrees of freedom and *P* value noted<br>*Give P values as exact values whenever suitable.*
[x]	[ ]	For Bayesian analysis, information on the choice of priors and Markov chain Monte Carlo settings
[x]	[ ]	For hierarchical and complex designs, identification of the appropriate level for tests and full reporting of outcomes
[x]	[ ]	Estimates of effect sizes (e.g. Cohen's *d*, Pearson's *r*), indicating how they were calculated

Our web collection on statistics for biologists contains articles on many of the points above.

Software and code

Policy information about availability of computer code

Data collection	Agilent BioTek Gen5 3.12 for analysis of absorbence assays (activity and BCA protein concentration), Bio-Rad CFX Manager 2.1 (software to import data and export to excel) , CFX Maestro for RT-PCR analysis of differential scanning fluorimetry (DSF, melting temperature determination), XDS (BUILT=20230630) to process the diffraction data.
Data analysis	python 3.14.0, numba 0.63.0b1, pandas 2.3.3, evcouplings 0.2.1, biopython 1.86, seaborn 0.13.2, openpyxl 3.1.5, igraph 1.0.0, scikit-learn 1.7.2, jupyter 1.1.1, numpy 2.3.5, PyMOL 2.5.2, AMoRe CCP4 v9.0, COOT 0.9.8.95 EL, BUCCANEER CCP4 v9.0, REFMAC 5.8.0430, AlphaFold 3.<br><br>Custom data analysis software will be available at https://github.com/gauthierscience/petase-protein-design [DOI: 10.5281/zenodo.22074022]

For manuscripts utilizing custom algorithms or software that are central to the research but not yet described in published literature, software must be made available to editors and reviewers. We strongly encourage code deposition in a community repository (e.g. GitHub). See the Nature Portfolio guidelines for submitting code & software for further information.

Data

Policy information about availability of data

All manuscripts must include a data availability statement. This statement should provide the following information, where applicable:

- Accession codes, unique identifiers, or web links for publicly available datasets
- A description of any restrictions on data availability
- For clinical datasets or third party data, please ensure that the statement adheres to our policy

There are no restrictions on data availability. All source data used for figures in this study are provided in the Source Data file provided by the journal. All code, raw and processed data are available at https://github.com/gauthierscience/petase-protein-design [DOI: 10.5281/zenodo.22074022], and model files and associated EVcouplings output are available at https://doi.org/10.5281/zenodo.17957682.

AddGene accession codes generated by this study are 257140 - 257223.

PDB files from crystal structures are available under PDB codes: 9PM1 [https://doi.org/10.2210/pdb9PM1/pdb] (TfCut2_EV2) and 9PM2 [https://doi.org/10.2210/pdb9PM2/pdb] (TfCut2_EV3)

Research involving human participants, their data, or biological material

Policy information about studies with human participants or human data. See also policy information about sex, gender (identity/presentation), and sexual orientation and race, ethnicity and racism.

Reporting on sex and gender	N/A
Reporting on race, ethnicity, or other socially relevant groupings	N/A
Population characteristics	N/A
Recruitment	N/A
Ethics oversight	N/A

Note that full information on the approval of the study protocol must also be provided in the manuscript.

Field-specific reporting

Please select the one below that is the best fit for your research. If you are not sure, read the appropriate sections before making your selection.

☒ Life sciences ☐ Behavioural & social sciences ☐ Ecological, evolutionary & environmental sciences

For a reference copy of the document with all sections, see nature.com/documents/nr-reporting-summary-flat.pdf

Life sciences study design

All studies must disclose on these points even when the disclosure is negative.

Sample size	We chose the number of designs to sample based on experimental tractability and resource constraints. A small number of starting enzymes (n=8) were used as parent enzymes to test feasibility in different starting backgrounds, each of which was used to generate 7-22 sub-designs. Sample size for the studies of diversity,. iccg-addition, and iterative design were generally limited to approx. 200 enzymes (2 x 96-well plates worth) plus controls. The number of designs per sample set (generally 10-20) was selected to maximize the number of sample sets while still testing a sufficient number of samples in each.
Data exclusions	No data were excluded - all tested designs are reported with all measured data.
Replication	The amount of protein acquired from high throughput purification determined the number of replicates we were able to profile during activity testing. Sometimes replicates were unable to be tested due to normal issues with purification. A validation round was performed on the top approximately 80 variants.
Randomization	No randomization was performed during characterization as samples were not allocated into groups because each sample represented a distinct group itself. Variants were evaluated under predefined assay conditions, with the number of conditions determined by available protein yield. For the Validation Set, designs and their corresponding parent enzymes were re-expressed, purified, and assayed in the same experimental run to control for batch-dependent variation.
Blinding	The assays performed in this manuscript yielded objective measurements such as thermostability, crystallography, and PET-depolymerization activity. The outcomes of these assays are not subject to subjective interpretation. Thus, blinding was not necessary.

Reporting for specific materials, systems and methods

We require information from authors about some types of materials, experimental systems and methods used in many studies. Here, indicate whether each material, system or method listed is relevant to your study. If you are not sure if a list item applies to your research, read the appropriate section before selecting a response.

Materials & experimental systems

n/a	Involved in the study
☒	☐ Antibodies
☒	☐ Eukaryotic cell lines
☒	☐ Palaeontology and archaeology
☐	☒ Animals and other organisms
☒	☐ Clinical data
☒	☐ Dual use research of concern
☒	☐ Plants

Methods

n/a	Involved in the study
☒	☐ ChIP-seq
☒	☐ Flow cytometry
☒	☐ MRI-based neuroimaging

Animals and other research organisms

Policy information about studies involving animals; ARRIVE guidelines recommended for reporting animal research, and Sex and Gender in Research

Laboratory animals	The study did not involve laboratory animals.
Wild animals	The study did not involve wild animals.
Reporting on sex	The study did not involve any animals.
Field-collected samples	The study did not involve any field collected samples.
Ethics oversight	No ethical guidance or approval was required as all experiments solely involved in vitro assays and generation of reagents from lab bacteria

Note that full information on the approval of the study protocol must also be provided in the manuscript.

Plants

Seed stocks	The study did not involve plants.
Novel plant genotypes	The study did not involve plants.
Authentication	The study did not involve plants.
