# Supplementary material for: Iterative computational and rational design generates diverse PET hydrolases for plastic depolymerization
Source: Nat Commun. 2026 Nov 3;17:11371. doi: 10.1038/s41467-026-78471-3 (PMC13731650; doi:10.1038/s41467-026-78471-3)
Supplement: Supplementary file 3 [file 41467_2026_78471_MOESM4_ESM.pdf]

nature portfolio

Peer Review File

Iterative computational and rational design generates diverse PET hydrolases for plastic depolymerization

Corresponding Author: Dr Nicholas Gauthier

This file contains all reviewer reports in order by version, followed by all author rebuttals in order by version.

Version 0:

Reviewer comments:

Reviewer #1

(Remarks to the Author)
This paper describes a major effort aimed at producing a variety of very efficient PET-hydrolyzing enzymes. This work is very important, both for its science and also for potential applications. The paper is very clearly written and the results are noteworthy.

A point that should be addressed in a few words is the ultimate aim of the approach chosen by the authors - is it just making a very large number of variants of the enzyme, or also choosing the best one? I am not sure that this was stated very clearly.

The term "primary sequence" should probably not be used - it is either "sequence" or "primary structure". I am also not sure that "average sequence" makes much sense.
"

(Remarks on code availability)

Reviewer #2

(Remarks to the Author)
This manuscript presents a promising and well-integrated strategy for engineering PET-degrading enzymes from the perspectives of protein engineering and computational design. By leveraging evolution-informed sequence modeling, the authors systematically explore a broad and otherwise inaccessible sequence space, yielding a large number of functional and improved variants.
However, from a chemical and materials science perspective, several additional clarifications would further strengthen the manuscript. First, the present study primarily relies on endpoint assays based on product formation after 48 hours of reaction. While this approach is sufficient for comparative screening, the absence of kinetic analyses, such as initial rate measurements or apparent kinetic parameters, limits deeper interpretation of the reported activity improvements. As a result, it remains difficult to distinguish whether the observed enhancements reflect increased intrinsic catalytic efficiency or instead arise from improved enzyme stability, prolonged lifetime, or increased residence time on the PET surface. For clarity, it would be helpful if the authors could more explicitly define in what sense certain variants are described as performing competitively with LCC-ICCG. Including time-course measurements of PET degradation, even for a limited set of representative enzymes, would further strengthen the interpretation.
Second, from a polymer chemistry perspective, additional characterization of the PET substrate during degradation would be beneficial. Although soluble products (TPA, MHET, and BHET) are quantified, information on changes in polymer properties, such as molecular weight, crystallinity, chain scission patterns, end-group formation, or the production of water-insoluble oligomers, is not provided. Even limited tracking of crystallinity during enzymatic degradation could offer valuable mechanistic insight into how the engineered enzymes act at the polymer-chain level.
Overall, this manuscript presents a compelling demonstration of EVdesign as a powerful tool for enzyme engineering. Addressing the points above, even in a limited manner, would further enhance the chemical interpretation and practical relevance of the study and would, in this reviewer’s view, strengthen the manuscript for publication. In particular, this reviewer would like to confirm whether the authors could provide time-course data for enzymatic PET degradation and corresponding changes in PET substrate crystallinity, at least for representative enzymes, to further support the chemical

interpretation of the reported performance improvements. I support publication of this manuscript after minor revision.

(Remarks on code availability)

Reviewer #3

(Remarks to the Author)
In this study, the authors apply EVdesign to design diverse PETases, demonstrating functional improvements across a range of temperatures and pH levels over several iterations. The extensive functional data generated provide a useful foundation for the field to analyze activity determinants in PETases and supporting future engineering efforts. I have included a few points below for clarification, primarily regarding the design methodology's underlying rationale.

Lines 84-86: “unlikely to emerge “ typically refers to epistatic mutations, not the simple accumulation of many mutations. The method and data presented do not support this claim.
Line 99: The mutational load is not an engineering goal; the change in activity under various conditions is. Dozens of surface mutations can be introduced without affecting activity, while <4 strategic active site mutations can induce dramatic activity changes. Clarify why the diversity generated by applying EVdesign iteratively is expected to generate functional benefits, and not just explore the natural sequence space.
Line 109: HT usually refers to thousands-millions variants. In this study, there are <1000.
Line 123: an all vs all rmsd/ seqid comparison would be a more intuitive way to compare sequence diversity. Active site composition would hint at functional diversity.
Line 127: The use of multiple MSA depths is mentioned but not justified/ explained.
Line 130: elaborate on what added value the EVcouplings model has over simple contact maps similar to those produced by e.g., AF
Line 133: Briefly explain what those metrics assess and why they are predictive of better fitness.
Lines 186-188 : beta-lactamases are used extensively in protein design method development and are known to be "evolvable". The sequence sampling is random and selection is controlled by EVH and the sequence similarity constraints -> similar to the previous comment, explain why EVH is expected to select for higher Tm
Lines 230-231: Is the parent enzyme active under the experimental conditions?
Line 264: functional novelty is not expected to be present at the natural diversity, i.e., evolutionary data. Unless fitness is mainly the similarity to the parent, it is not expected to drop that drastically.
Line 265: Why only 48% of the low diversity designs were purified (compared to 80% from med)? Is there a consistently bad mutation common to those failing to be purified?
Lines 303-307: evolutionary similarity is good for assessing thermostability, but may be opposite to functional novelty, especially in active site regions. Suggest alternative ranking schemes that will result in fewer non-expressed proteins in the high diversity sets.
Line 348: AF is known to be robust to point mutations and is not a good indicator of mutant fitness
Figure 4: Panel subtitles are distracting
Line 525: Seems like the main contribution is generating diversity. Elaborate on why EVdesign sequences are expected to manifest more functional gains than simple methods like consensus design or metagenomic mining.
Lines 537-539: Many design campaigns start by stabilizing the parent enzyme with methods like PROSS or consensus design to allow a higher mutational load. Alternatively, a single detrimental mutation can abolish activity/stability. Explore whether the “non-evolvable” parents had such mutations.
Lines 569-579: This paragraph argues that EVdesign by itself is limited in finding functionally superior variants as it is constrained by evolutionary information, while functional novelty arises by stepping away from conservation. The "combination" of methods is possible in any enzyme engineering/design method by simply introducing the "rational" mutation into the parent's background.
Line 582: refer to % mutations. Homologs can have <50% identity and still adopt the same structure and function
Lines 584-587: compare to equivalent, relevant methods - i.e., other enzyme design methods (e.g., from Arnold and Fleishman labs, protein LLMs)
Lines 601-603: The synthesis cost of full-length enzymes would be impractical for medium to high-throughput synthesis. The number tested here (<1000) is not a large enough dataset for training large models, going beyond the protein family.
Line 654: Starting from a random sequence would make most of the sampling time be spent on "converging" back into a PETase

(Remarks on code availability)

Version 1:

Reviewer comments:

Reviewer #1

(Remarks to the Author)
All my original concerns were fully answered.

(Remarks on code availability)

Reviewer #2

(Remarks to the Author)
The authors properly responded to the comments raised by this reviewer. Regarding the evaluation of enzyme activity using small-scale 48-hour assays, this reviewer would like to clarify that the previous comment did not intend to request time-course measurements for all specimens. Rather, it was intended to suggest that representative enzymes, such as TfCut2-ICCG and LCC-ICCG, could be analyzed to provide additional mechanistic insight.

A 48-hour reaction period is relatively long for enzyme activity measurements, and time-course measurements can provide fundamental information regarding how the enzymatic activity develops and whether it is sustained over time during the reaction. In addition, such experiments are not considered to be experimentally difficult to perform.

(Remarks on code availability)

Reviewer #3

(Remarks to the Author)
I thank the authors for their detailed and considered responses to my initial comments. Each of the points raised has been addressed, either through revisions to the manuscript or through clear and well-reasoned clarification. The manuscript is improved, and I recommend acceptance for publication

(Remarks on code availability)

Version 2:

Reviewer comments:

Reviewer #2

(Remarks to the Author)
I appreciate the authors' detailed response and understand their explanation that these experiments are outside the scope of the present study. However, my concern still remains.

I did not intend to request a detailed mechanistic study. My suggestion was only to perform a simple time-course analysis using one or two representative enzymes, such as TfCut2-ICCG and LCC-ICCG. Since the enzyme activity was evaluated only after a relatively long reaction time (48 h), I think such data would provide useful information about the development and persistence of enzyme activity and would strengthen the manuscript.

The authors have clearly explained why they do not wish to perform these experiments. Therefore, I do not think further discussion on this point would be productive, and I leave the final decision to the editor.

AI use: An AI tool was used to improve the English wording of this review. All scientific judgments and opinions are my own.

(Remarks on code availability)

Open Access This Peer Review File is licensed under a Creative Commons Attribution 4.0 International License, which permits use, sharing, adaptation, distribution and reproduction in any medium or format, as long as you give appropriate credit to the original author(s) and the source, provide a link to the Creative Commons license, and indicate if changes were made.
In cases where reviewers are anonymous, credit should be given to 'Anonymous Referee' and the source.
The images or other third party material in this Peer Review File are included in the article's Creative Commons license, unless indicated otherwise in a credit line to the material. If material is not included in the article's Creative Commons license and your intended use is not permitted by statutory regulation or exceeds the permitted use, you will need to obtain permission directly from the copyright holder.
To view a copy of this license, visit https://creativecommons.org/licenses/by/4.0/

Iterative computational and rational design generates diverse PET hydrolases for plastic depolymerization

Point-by-point responses to reviewers' comments:

Thank you to all of the reviewers for your constructive and insightful comments. Below please find a point-by-point response to each individual reviewer.

Reviewer #1 (Remarks to the Author):

This paper describes a major effort aimed at producing a variety of very efficient PET-hydrolyzing enzymes. This work is very important, both for its science and also for potential applications. The paper is very clearly written and the results are noteworthy.

A point that should be addressed in a few words is the ultimate aim of the approach chosen by the authors - is it just making a very large number of variants of the enzyme, or also choosing the best one? I am not sure that this was stated very clearly.

We thank the reviewer for the positive assessment of our work and for this helpful suggestion. We agree that the ultimate objective could be stated more explicitly and have revised the Introduction (Paragraph 5) to clarify this point.

The term "primary sequence" should probably not be used - it is either "sequence" or "primary structure". I am also not sure that "average sequence" makes much sense.

We thank the reviewer for these helpful clarifications. We have revised all instances of “primary sequence” to “sequence” and the instance of “average sequence” to “mean performance of the design set”.

Reviewer #2 (Remarks to the Author):

This manuscript presents a promising and well-integrated strategy for engineering PET-degrading enzymes from the perspectives of protein engineering and computational design. By leveraging evolution-informed sequence modeling, the authors systematically explore a broad and otherwise inaccessible sequence space, yielding a large number of functional and improved variants.

However, from a chemical and materials science perspective, several additional clarifications would further strengthen the manuscript. First, the present study primarily relies on endpoint assays based on product formation after 48 hours of reaction. While this approach is sufficient for comparative screening, the absence of kinetic analyses, such as initial rate measurements or apparent kinetic parameters, limits deeper interpretation of the reported activity improvements. As a result, it remains difficult to distinguish whether the observed enhancements reflect increased intrinsic catalytic efficiency or instead arise from improved enzyme stability, prolonged lifetime, or increased residence time on the PET surface. For clarity, it would be helpful if the authors could more explicitly define in what sense certain variants are described as performing competitively with LCC-ICCG. Including time-course measurements of

PET degradation, even for a limited set of representative enzymes, would further strengthen the interpretation.

We thank the reviewer for this feedback and agree that time-resolved kinetic analyses would provide additional mechanistic insight. This study was designed as a large-scale comparative evaluation, encompassing over 600 variants processed through the purification pipeline and approximately 18,000 depolymerization reactions spanning enzyme, temperature, pH, and substrate conditions (with replicate measurements). As such, the experimental framework prioritizes breadth of screening over detailed enzymology of individual variants, and time-course kinetic analyses were not included here. However, we have incorporated these ideas into a new Paragraph 7 in the Discussion, which details how future mechanistic studies will be applied to understand the relative contributions of catalytic turnover, enzyme stability, and substrate interaction dynamics for high-performing variants.

To the reviewer's second point, we have also emphasized in the Results section under subheading "Iterative EVdesign leads to variants with further increases in activity" Paragraph 5 that "competitive with LCC-ICCG" refers specifically to equal or greater 48-hour product formation under identical enzyme loading, buffer, substrate, and temperature conditions.

Second, from a polymer chemistry perspective, additional characterization of the PET substrate during degradation would be beneficial. Although soluble products (TPA, MHET, and BHET) are quantified, information on changes in polymer properties, such as molecular weight, crystallinity, chain scission patterns, end-group formation, or the production of water-insoluble oligomers, is not provided. Even limited tracking of crystallinity during enzymatic degradation could offer valuable mechanistic insight into how the engineered enzymes act at the polymer-chain level.

Indeed, characterization of polymer-property evolution during degradation could provide valuable mechanistic insight, but as stated above, the primary objective of this study was large-scale comparative analysis of enzyme variants under standardized conditions. To enable consistent evaluation, we focused on quantification of soluble depolymerization products using pre-characterized PET substrates. More detailed characterization of polymer-property changes during enzymatic degradation typically requires additional analytical workflows (e.g., GPC, DSC, XRD) that would constitute a substantial extension of the experimental scope. We agree that such analyses would be valuable for deeper mechanistic interpretation, and we have incorporated these ideas into the same new "future directions" Paragraph 7 in the Discussion as mentioned in the previous comment.

Overall, this manuscript presents a compelling demonstration of EVdesign as a powerful tool for enzyme engineering. Addressing the points above, even in a limited manner, would further enhance the chemical interpretation and practical relevance of the study and would, in this reviewer's view, strengthen the manuscript for publication. In particular, this reviewer would like to confirm whether the authors could provide time-course data for enzymatic PET degradation and corresponding changes in PET substrate crystallinity, at least for representative enzymes, to further support the chemical interpretation of the reported performance improvements. I support publication of this manuscript after minor revision.

We thank the reviewer for their constructive evaluation and supportive recommendation. While time-resolved degradation data and polymer-property characterization would provide additional

mechanistic insight, the primary contribution of this work is the generation and dissemination of a large, systematically evaluated dataset of PET hydrolase variants across diverse conditions, a release of the open-source software and protocols to apply the methodology to different proteins of interest, together with a demonstration of the effectiveness of EVcouplings-based design for this problem. We have clarified the interpretation of the endpoint assays and explicitly acknowledged these considerations in the revised manuscript. Building on the results presented in this manuscript, ongoing work is focused on detailed mechanistic characterization of selected high-performing variants, which will serve as starting points for further engineering and evolution under process-relevant conditions.

Reviewer #3 (Remarks to the Author):

In this study, the authors apply EVdesign to design diverse PETases, demonstrating functional improvements across a range of temperatures and pH levels over several iterations. The extensive functional data generated provide a useful foundation for the field to analyze activity determinants in PETases and supporting future engineering efforts.

We thank the reviewer for their positive assessment.

I have included a few points below for clarification, primarily regarding the design methodology's underlying rationale.

Lines 84-86: “unlikely to emerge “ typically refers to epistatic mutations, not the simple accumulation of many mutations. The method and data presented do not support this claim.

We have revised the text, including removing the “unlikely to emerge” statement, to more accurately describe the scope of the method. Our intention was to convey that EVdesign enables coordinated sampling of multiple combinations of mutations guided by evolutionary constraints, rather than simple accumulation of independent substitutions.

Line 99: The mutational load is not an engineering goal; the change in activity under various conditions is. Dozens of surface mutations can be introduced without affecting activity, while <4 strategic active site mutations can induce dramatic activity changes. Clarify why the diversity generated by applying EVdesign iteratively is expected to generate functional benefits, and not just explore the natural sequence space.

We agree that we are likely not sampling sequences that arise through natural evolution. In the past, we have hypothesized about “why” we see functional benefits using EVdesign (see Fram et al, 2024). In short, we believe that functional benefits may arise because the EVcouplings predicted fitness formulation, which is the summation of pairwise amino acid “couplings” and positional conservation, combines constraints found across distinct proteins in the multiple sequence alignment into one distinct design. The natural sequences likely only need a minimum number of constraints to satisfy evolutionary pressure. Although this is speculation, we agree that the conceptual details of how the EVdesign methodology works were quite sparse in the main text, and we have now added more detail into the introduction about the method.

Line 109: HT usually refers to thousands-millions variants. In this study, there are <1000.

We appreciate the reviewer's comment regarding terminology. While "high-throughput" can refer to very large variant libraries in some contexts, our use of the term refers specifically to semi-automated expression, purification, and multi-condition biochemical characterization of enzyme variants. Purified enzyme screening across multiple substrates, temperatures, and pH conditions is substantially more resource-intensive than growth-based or crude-lysate screens. In total, this study involved characterization of >600 variants across >18,000 depolymerization reactions—a scale that necessitates automation and represents high-throughput analysis for purified enzyme assays.

Line 123: an all vs all rmsd/ seqid comparison would be a more intuitive way to compare sequence diversity. Active site composition would hint at functional diversity.

We have added an additional table to the supplement (Supplementary Table S1) that contains both the all vs all RMSD and the all vs all sequence identity for the eight scaffolds used for design generation and testing.

Line 127: The use of multiple MSA depths is mentioned but not justified/ explained.

The justification was described later in the paragraph, but it was confusing as written. We have rearranged the paragraph to better explain why we generate models from several MSAs. In short, each MSA is used to create a distinct model, and the "best" model was selected as the one that had the highest predicted accuracy and coverage.

Line 130: elaborate on what added value the EVcouplings model has over simple contact maps similar to those produced by e.g., AF

The EVcouplings model can be probed for contact maps (primarily for validation and use in downstream structure prediction), but it is the EVcouplings model formulation (sum of pairwise interactions and position conservation) itself that is used as a metric to generate sequences. AlphaFold can also be probed for contacts, but it is not an energy function that can be used to generate sequences.

Line 133: Briefly explain what those metrics assess and why they are predictive of better fitness.

We agree that the amount of background on the predicted fitness metric was insufficient, and have added additional detail to the introduction that describes the predicted fitness score calculation (pairwise couplings and sitewise conservation) and have also cited additional papers that have used this model to understand different aspects of biology (structure and stability prediction, mutational effects prediction, etc).

Lines 186-188 : beta-lactamases are used extensively in protein design method development and are known to be "evolvable". The sequence sampling is random and selection is controlled by EVH and the sequence similarity constraints -> similar to the previous comment, explain why EVH is expected to select for higher Tm

Similar to the comment above (see Line 99), we can only speculate the mechanism for "why" sequences with a high EVH tend to have increased stability, however there is quite a bit of

empirical evidence for this correlation. We have added citations and expanded upon how the method works in the introduction.

Lines 230-231: Is the parent enzyme active under the experimental conditions?

All parent enzymes included in this study have been previously demonstrated to exhibit PET hydrolysis activity under similar assay conditions (Erickson et al., Nat. Comm. 2022). We have added a statement to the manuscript to clarify this point.

Line 264: functional novelty is not expected to be present at the natural diversity, i.e., evolutionary data. Unless fitness is mainly the similarity to the parent, it is not expected to drop that drastically.

The MSAs used in this work include a broad range of hydrolase sequences and therefore encompass natural functional diversity on multiple substrates in various conditions. In our framework, sequence identity constraints to the parent were held constant across all diversity levels, while diversity was modulated by adjusting Gibbs sampling parameters (i.e. convergence rate and number of iterations). As these constraints were relaxed, the sampling explored regions of sequence space that are less optimal with respect to the predicted fitness (evolutionary hamiltonian, EVH). We therefore are not surprised that the EVH score is lower as the stringency of sampling is reduced.

Line 265: Why only 48% of the low diversity designs were purified (compared to 80% from med)? Is there a consistently bad mutation common to those failing to be purified?

We hesitate to overinterpret differences in purification success across design sets. Expression and solubility in *E. coli* are influenced by many factors, including folding efficiency, aggregation propensity, codon usage, and sensitivity to expression conditions. Expression and purification were not systematically optimized for individual variants, and modest changes in conditions may have enabled successful purification of additional designs. Moreover, given the small-scale purification format used here, some variants may have fallen below detection thresholds, and the observed differences may therefore reflect experimental variability rather than systematic effects of design diversity.

Lines 303-307: evolutionary similarity is good for assessing thermostability, but may be opposite to functional novelty, especially in active site regions. Suggest alternative ranking schemes that will result in fewer non-expressed proteins in the high diversity sets.

While additional filtering or ranking strategies could potentially improve expression success, many available approaches are based on evolutionary or structural likelihood, which are conceptually similar to the EVcouplings framework used here and would likely bias sampling toward less diverse sequences. We also note that even in the most diverse set, which had particularly poor EVH scores (also noted by the reviewer above), a substantial fraction of designs (~31%) expressed and purified. We consider this a relatively high hit rate for a single-round design approach at this level of sequence divergence. However, for protein design problems in which one requires a higher expression “hit rate”, we’d recommend being more stringent with the Gibbs sampling parameters (increasing the number of iterations and/or

increasing the convergence rate) rather than introducing the complexity of additional ranking schemes. We have now added this recommendation to the Discussion.

Line 348: AF is known to be robust to point mutations and is not a good indicator of mutant fitness

Molecular dynamics using AMBER post prediction ensures side chain positions are physically reasonable, and indeed we observe the disulfide contact and isoleucine and glycine positions to be similarly oriented in LCC-ICCG and TfCut2-ICCG. Robustness of the tertiary structure to mutations is not of concern as we know the protein folds and functions experimentally. We added more detail about how the side chains are positioned in the prediction.

Figure 4: Panel subtitles are distracting

We simplified the subtitles in this figure, including the removal of all the phrasing that included interpretation.

Line 525: Seems like the main contribution is generating diversity. Elaborate on why EVdesign sequences are expected to manifest more functional gains than simple methods like consensus design or metagenomic mining.

Broadly speaking, we view EVdesign as part of a larger toolkit of enzyme engineering strategies. Approaches such as metagenomic mining and consensus design are powerful and widely used for enzyme discovery and optimization, but are substantially different from the design methodology presented in this work and a fair comparison across these and other enzyme engineering strategies would require a broader and more detailed discussion than is feasible within this manuscript. To discuss briefly here, metagenomic mining enables the identification of naturally occurring proteins with diverse and unique activity profiles; indeed, many of the starting scaffolds used in this study were identified through our prior metagenomic efforts (see Erickson et al., 2022). EVdesign builds upon these scaffolds by enabling further exploration of sequence space to improve properties such as activity, stability, and substrate preference. Empirically, we observed higher performance from the EVdesign variants than in our PETase metagenomic efforts (Erickson et al., 2022 and Norton-Baker et al., 2025). Consensus-based methods, which typically consider positions independently and favor conserved residues, can be particularly effective for stabilizing proteins. EVdesign differs in that it incorporates positional conservation while also leveraging co-evolutionary constraints to model relationships between residues (i.e., couplings). This incorporation of epistatic information to the design strategy potentially allows the generation of coordinated multi-site mutation combinations, which we speculate may lead to additional functional gains beyond conservation-only methods.

Lines 537-539: Many design campaigns start by stabilizing the parent enzyme with methods like PROSS or consensus design to allow a higher mutational load. Alternatively, a single detrimental mutation can abolish activity/stability. Explore whether the “non-evolvable” parents had such mutations.

The intent of the referenced sentence was to discuss the natural scaffolds, which do not contain mutations, rather than the iteratively designed parents. This was unclear as written, and we

have updated the text accordingly. The reviewer's comment also raises an interesting point about pre-stabilizing the sequences to enable mutational tolerance, and we have added this possible solution to poor performing scaffolds to the main text.

Lines 569-579: This paragraph argues that EVdesign by itself is limited in finding functionally superior variants as it is constrained by evolutionary information, while functional novelty arises by stepping away from conservation. The "combination" of methods is possible in any enzyme engineering/design method by simply introducing the "rational" mutation into the parent's background.

There is a limitation with the EVdesign methodology in that it will not score novel substitutions positively that do not exist or are rare in the multiple sequence alignment. We also agree that many methods may benefit from combining rational design, e.g., starting with TfCut2-ICCG rather than TfCut2. The interesting / unexpected result is that the EVdesign methodology is tolerant to these unnatural mutations even though they are nearly non-existent in the MSA, and many of the resulting designs exhibited further increases in performance over the rationally-designed parent (i.e., TfCut2-ICCG). We added additional context in the Results section in the pointer to the logo plot of the natural homologs (Figure 4E).

Line 582: refer to % mutations. Homologs can have <50% identity and still adopt the same structure and function

We have updated the text with the % mutations (>20%).

Lines 584-587: compare to equivalent, relevant methods - i.e., other enzyme design methods (e.g., from Arnold and Fleishman labs, protein LLMs)

We agree that comparison to other enzyme engineering approaches is valuable; however, such comparisons would require more extensive discussion (and analysis / benchmarking) than can be accommodated within this manuscript, as methods including directed evolution (e.g., Arnold lab), structure-based design (e.g., PROSS/FuncLib), and emerging machine learning–based approaches differ substantially in their objectives, sampling strategies, and evaluation metrics. A comprehensive comparison across these approaches would therefore require a more detailed treatment and is more appropriately addressed in dedicated reviews.

We note that the Introduction references many of these methods and we have also revised paragraph 3 of the introduction to more clearly describe how EVdesign operates to help differentiate it from other enzyme engineering approaches.

Lines 601-603: The synthesis cost of full-length enzymes would be impractical for medium to high-throughput synthesis. The number tested here (<1000) is not a large enough dataset for training large models, going beyond the protein family.

We agree that the amount of data we generated is insufficient to train new generative models that go beyond the protein family. Rather our intention was to point out that this methodology is a means towards generating additional / augmenting the data needed to train predictors and machine-learning models. We have updated the text to more realistically reflect the utility of these data.

Line 654: Starting from a random sequence would make most of the sampling time be spent on "converging" back into a PETase

Indeed there are additional strategies one could apply to reduce the sampling time, e.g., starting with the parent sequence or closer to the parent sequence. However, the decision to begin with a random sequence was purposeful to avoid local minima and (reasonably) maximize exploration of the search space.

Iterative computational and rational design generates diverse PET hydrolases for plastic depolymerization

Point-by-point responses to reviewers' comments:

Thank you to all the reviewers for their careful evaluation of the revised manuscript and thoughtful feedback. Below please find a point-by-point response to each individual reviewer.

Reviewer #1 (Remarks to the Author):

All my original concerns were fully answered.

Reviewer #2 (Remarks to the Author):

The authors properly responded to the comments raised by this reviewer. Regarding the evaluation of enzyme activity using small-scale 48-hour assays, this reviewer would like to clarify that the previous comment did not intend to request time-course measurements for all specimens. Rather, it was intended to suggest that representative enzymes, such as TfCut2-ICCG and LCC-ICCG, could be analyzed to provide additional mechanistic insight.

A 48-hour reaction period is relatively long for enzyme activity measurements, and time-course measurements can provide fundamental information regarding how the enzymatic activity develops and whether it is sustained over time during the reaction. In addition, such experiments are not considered to be experimentally difficult to perform.

We apologize if our previous response was unclear. We did understand that the intent was to suggest these experiments for only a small, representative set of enzymes rather than the entire screening library. We respectfully maintain that such experiments fall outside the scope of the current study. The central objective of this work is the development and validation of an iterative computational and rational design framework for generating diverse, high-performing PET hydrolases. While time-course measurements on a limited number of variants could provide additional descriptive data, these measurements will not provide sufficient mechanistic insight into the origins of the observed performance differences.

As the reviewer noted in their original comments, PET depolymerization is influenced by numerous interconnected factors, including intrinsic catalytic activity, enzyme stability, productive interaction with the polymer surface, substrate accessibility, and reaction-driven changes in pH. A limited time-course analysis performed on only a few representative enzymes under screening conditions would not allow these contributions to be disentangled. Consequently, any observed trends would be difficult to interpret mechanistically and may not generalize across the broader set of designs generated by the workflow.

Accordingly, while we agree that a rigorous mechanistic investigation of the factors governing enzyme performance would be valuable, such a study would undoubtedly require substantially expanded experimentation, including kinetic measurements across multiple variants, polymer

characterization, structural analysis, and ideally pH-controlled depolymerization experiments capable of separating catalytic efficiency and enzyme stability from pH-driven inactivation effects. We view this as a distinct future research direction focused on mechanistic understanding rather than enzyme discovery and design.

Reviewer #3 (Remarks to the Author):

I thank the authors for their detailed and considered responses to my initial comments. Each of the points raised has been addressed, either through revisions to the manuscript or through clear and well-reasoned clarification. The manuscript is improved, and I recommend acceptance for publication.

Iterative computational and rational design generates diverse PET hydrolases for plastic depolymerization

Point-by-point responses to reviewers' comments:

Thank you to all the reviewers for their careful evaluation of the revised manuscript and thoughtful feedback. Below please find a point-by-point response to each individual reviewer.

Reviewer #2 (Remarks to the Author):

I appreciate the authors' detailed response and understand their explanation that these experiments are outside the scope of the present study. However, my concern still remains.

I did not intend to request a detailed mechanistic study. My suggestion was only to perform a simple time-course analysis using one or two representative enzymes, such as TfCut2-ICCG and LCC-ICCG. Since the enzyme activity was evaluated only after a relatively long reaction time (48 h), I think such data would provide useful information about the development and persistence of enzyme activity and would strengthen the manuscript.

The authors have clearly explained why they do not wish to perform these experiments. Therefore, I do not think further discussion on this point would be productive, and I leave the final decision to the editor.

AI use: An AI tool was used to improve the English wording of this review. All scientific judgments and opinions are my own.

We thank the reviewer for clarifying their suggestion. We have revised the Discussion to more explicitly describe this future research direction.
